# mRNA ratios of *AR* to *ESR1* and *PGR* distinguish breast cancer subtypes based on public datasets and experimental models

**DOI:** 10.1038/s41598-025-06856-3

**Published:** 2025-07-01

**Authors:** Diego Prieto, Milena Rondón-Lagos, Paola Cruz-Tapias, Andrés Rincón-Riveros, Wilson Rubiano, Jairo De la Peña, Elizabeth Vargas, Victoria E. Villegas, Nelson Rangel

**Affiliations:** 1 School of Biological Sciences, Universidad Pedagógica y Tecnológica de Colombia, 150003 Tunja, Colombia; 2 Department of Cardiology, Fundación Cardioinfantil, LaCardio, 1113111 Bogota, Colombia; 3 Bacteriology Program, Faculty of Health Sciences, Universidad Colegio Mayor de Cundinamarca, 110311 Bogotá, Colombia; 4 Hospital Universitario Mayor-Méderi, Universidad del Rosario, 111411 Bogotá, Colombia; 5 Centro de Investigaciones en Microbiología y Biotecnología-UR (CIMBIUR), Facultad de Ciencias Naturales, Universidad del Rosario, Cra. 24, # 63C-69, 111221 Bogotá, Colombia; 6 Departamento de Nutrición y Bioquímica, Facultad de Ciencias, Pontificia Universidad Javeriana, Cra. 7 # 40-62, 110231 Bogotá, Colombia

**Keywords:** Breast cancer, Molecular subtypes, Androgen receptor, Estrogen receptor, Progesterone receptor, Meta-analysis, Biochemistry, Cancer, Computational biology and bioinformatics, Molecular biology, Biomarkers, Oncology

## Abstract

The role of the androgen receptor (AR) in breast cancer (BC) remains incompletely understood. Here, we conducted a meta-analysis of large-scale microarray transcriptomic datasets to evaluate whether the mRNA expression levels of the androgen receptor gene, relative to those of the estrogen receptor gene (*AR/ESR1* ratio) and the progesterone receptor gene (*AR/PGR* ratio), can help differentiate BC tumor subtypes. Additionally, we used qRT-PCR assays to assess the mRNA levels of the *AR/ESR1* and *AR/PGR* ratios in four cell lines representative of different BC subtypes (MCF7, BT474, MDA-MB453, and MDA-MB231), as well as in breast tissue from a small group of patients (11 cases) stratified by estrogen receptor (ER) status. Our results showed that higher *AR* gene expression relative to *ESR1* and *PGR* (≥ 2.0 and ≥ 1.54, respectively) were associated with BC patients classified under the Luminal B and HER2-enriched subtypes. Positive values of *AR/ESR1* and *AR/PGR* ratios were also observed in the ER-negative (ER-) cell line MDA-MB453, as well as in tumor tissue from ER- BC patients. Our findings confirm that higher or even positive *AR/ESR1* and *AR/PGR* ratios may be associated with BC cases exhibiting more aggressive clinical and biological features, leading to a worse prognosis.

## Introduction

Breast cancer (BC) remains the most diagnosed cancer among women worldwide, with approximately 2.3 million new cases. The disease also caused around 670,000 deaths globally. Projections by the International Agency for Research on Cancer (IARC) estimate that by 2040, BC cases could exceed 3 million annually, with over 1 million deaths each year, reflecting a significant increase in global burden^1,2^. BC is widely recognized as a hormone-dependent disease, as approximately 75% of breast tumors express estrogen receptors (ER) and progesterone receptors (PgR). These hormone receptors serve as valuable prognostic markers and are also predictive of treatment response^3^. Different BC subtypes have been identified, defined not only by the presence or absence of hormone receptors but also by varying expression levels of these receptors. Gene expression studies have facilitated the classification of four major intrinsic molecular subtypes: Luminal tumors (A and B), which account for approximately 70% of all cases and are characterized by low proliferation and high ER gene signature activity; Basal-like, or triple-negative breast cancer (TNBC), comprising about 20% of cases, characterized by high proliferation and the absence of ER, PgR, and HER2 expression; and finally, HER2-positive non-luminal cancers (HER2-enriched), defined by HER2 overexpression and the absence of both ER and PgR, indicating a distinct aggressive subtype that constitutes 10–15% of all BC cases^4–7^.

Notwithstanding, the role of the androgen receptor (AR), another important hormone receptor, has not been clearly defined in BC. AR is a nuclear receptor whose function is stimulated by testosterone or dihydrotestosterone. Several studies have shown that AR expression in ER positive (ER +, luminal) tumors is considered an independent factor associated with good prognostic features (*e.g.,* low grade, longer disease-free survival)^8–10^. However, in the ER negative (ER-) BC subgroup, the prognostic value of this marker is unclear, given that original investigations present conflicting results^11–13^. Consistent with the above, in vitro studies show that AR signaling inhibits the proliferation of ER + cells (*i.e.* MCF7 cell line) when induced by estrogens^14–16^, but other studies have shown that AR can promote the proliferation of ER- BC cells, particularly in cases classified as apocrine or luminal androgen receptor (LAR) molecular subtypes^17,18^. Therefore, several authors suggest that AR appears to have opposing effects on BC prognosis, depending on ER and/or PgR status or even on the relationship between their expression levels^19^. Indeed, recent research has established that BC cases with high AR levels relative to ER or PgR (*i.e.* AR/ER ≥ 2 and AR/PgR ≥ 1.54, respectively) are associated with aggressive clinical and biological features, poor prognosis^20–23^ and resistance to therapy^24^. These associations have been observed even in carcinoma in situ^25^ and in young premenopausal women with BC^26,27^.

It has been clearly reported that both ER and AR function as transcription factors, with each potentially mediating specific hormonally regulated transcriptional programs. Thus, the behavior of BC cells and the progression of tumor development can be modulated not only by receptor expression levels (at the genomic and/or proteomic level), but also by the specific patterns of receptor binding across the genome, which in turn alter gene expression profiles (cistrome rearrangements). These mechanisms may influence key tumor characteristics such as cellular proliferation, therapeutic resistance, and autonomous hormone responsiveness^28–30^. This additionally suggests the potential for therapeutic strategies targeting combined hormonal signaling pathways, since approaches correlating the relative levels of hormone receptors with treatment have shown that the AR/ER ratio is not always predictive of the benefit from extended endocrine therapy^31^.

Many studies have reported a high degree of BC cell heterogeneity, attributed to variations in the expression of classical markers (ER, PgR, HER2, Ki-67) and the presence of additional markers such as the AR^32^. However, these conventional markers are insufficient to fully capture the complexity of BC. Given that the prognostic value of the relative expression of hormone receptors remains unclear, this study aimed to evaluate the clinical relevance of AR expression levels relative to *ESR1* (*AR/ESR1* ratio) and *PGR* (*AR/PGR* ratio) by conducting a meta-analysis of large-scale microarray transcriptomic datasets. To further validate whether these ratios can differentiate BC subtypes, we assessed *AR/ESR1* and *AR/PGR* mRNA expression in four representative BC cell lines. Lastly, we applied the same evaluation to tumor tissues from a small cohort of BC patients to explore the ratios’ ability to distinguish between ER + and ER- cases.

## Materials and methods

### Microarray databases search strategy in the gene expression omnibus (GEO) repository

Microarrays containing data from patients with breast cancer (BC) were retrieved from the publicly accessible Gene Expression Omnibus (GEO) website https://www.ncbi.nlm.nih.gov/geo/. This international repository allows free querying, downloading, and analysis of genomic data. The search strategy included the terms (“Breast Neoplasms [Mesh]”) AND “Expression Profiling” AND (“Homo sapiens” [porgn:_txid9606]), with a deadline for inclusion of August 2020.

The inclusion criteria were the following: (I) Enrolled data were obtained from humans; (II) Microarray datasets with information on gene expression (mRNA) of AR (*AR*), ER (*ESR1*) and PgR (*PGR*); (III) The samples should not be from cell lines; (IV) Sufficient data for Odds Ratio calculation; (V) Patients with primary invasive BC with clinical information available; (VI) BC patients without treatment before sample collection.

### Data extraction

From the data sets filtered by the inclusion criteria, the following variables were extracted: GEO accession number, PubMed Identifier (PMID), age, histologic grade, tumor stage, tumor size (T), lymph nodal status (N), metastasis (M), and ER, PgR, and human epidermal growth factor receptor 2 (HER2) status. The model proposed by Carey et al.^33^ was followed to classify the surrogate subtypes by IHC as Luminal A (hormone receptor positive—HR +/HER2-), Luminal B (HR +/HER2 + and/or high Ki-67), HER2-enriched (hormone receptor negative—HR-/HER2 +), or TNBC (HR-/HER2-). The GEO2R tool (based on R language) integrated into NCBI^34^ was used to extract mRNA expression values of the *AR*, *ESR1*, and *PGR* genes. The expression values of the *AR/ESR1* and *AR/PGR* ratios were calculated using a quotient between the mRNA expression values of the receptors involved in each ratio. Microarray datasets were used where the intrinsic molecular subtypes (Luminal A, Luminal B, HER2-enriched, Basal-like, and Normal-like) had been defined using the classification algorithms reported by PAM50^4^, Hu et al.^35^, or Sørlie et al.^36^.

### Cell lines

MCF7 (ER +/HER2-), BT474 (ER +/HER2 +), MDA-MB453 (ER-/HER2 +), and MDA-MB231 (ER-/HER2-) cell lines were obtained from the American Type Culture Collection (ATCC). MCF7, MDA-MB453, and MDA-MB231 were cultured in RPMI-1640 medium (Sigma), while BT474 was cultured in DMEM medium (Sigma). All culture media were supplemented with 1X antibiotic–antimycotic solution (Sigma), 10% fetal bovine serum (FBS) (Sigma), and 2 mM L-glutamine (Invitrogen GmbH) and maintained at 37 °C and 5% CO_2_. The absence of mycoplasma contamination was confirmed by PCR.

### BC tumor tissue

4 ER +/PgR +/HER2- and 2 ER-/PgR-/HER2 + cases, as well as 5 fibroadenomas (FA) were chosen from patients who attended a mastology consultation at the Hospital Méderi in Bogotá, Colombia. All tissues were collected after patients had signed informed consent. The diagnosis was made by the attending physician based on physical examination, tests, and histopathological confirmation by biopsy. The study and the use of biological samples were approved by the Ethics Committee of the Universidad del Rosario.

### RNA extraction and sequencing (RNA-Seq)

Total RNA was isolated using the RNeasy kit (Qiagen, Germany), following the manufacturer’s instructions. RNA quantification and purity analysis were performed using the NanoDrop 2000 (ThermoScientifc. Wilmington, DE, USA). The preparation of RNA libraries and mRNA sequencing of cell lines were carried out by Novogene Corporation Inc. (Sacramento, CA, USA) on the Illumina HiSeq 2500 platform (paired-end 150-nucleotide read length).

### qRT-PCR

1 μg of total RNA (cell lines & tumor tissue) was used for complementary DNA (cDNA) synthesis with the High-Capacity Reverse Transcription Kit (Applied Biosystems, Foster City, CA, USA), following the manufacturer’s instructions. To evaluate mRNA levels, TaqMan® Gene Expression assays (Applied Biosystems, Foster City, CA, USA) for *AR* (Assay ID: Hs00171172_m1), *ESR1* (Assay ID: Hs00174860_m1), *PGR* (Assay ID: Hs01556702_m1) *GLI1* (Assay ID: Hs00171790_m1), and *GAPDH* (Assay ID: Hs02786624_g1) were run in triplicates following the manufacturer’s instructions on the LightCycler 96 real-time thermal cycler (Roche, Germany). Differences in gene expression (relative to the *GLI1* for which lower expression levels were observed) for *AR/ESR1* and *AR/PGR* ratios were calculated using the 2^-ΔΔ ^Ct method.

### Statistical analysis

For meta-Analysis, cases were grouped based on the previously established cutoff points for each ratio, 2.0 and 1.54 for AR/ER and AR/PgR, respectively^20,22^. For each GEO dataset, contingency tables were used to analyze the relationship between categories of the same clinicopathological variable as well as between molecular subtypes. The degree of association between the expression values of the ratios with the IHC-surrogate or intrinsic molecular subtypes and with the clinicopathological characteristics of BC patients was established using Odds Ratio with 95% confidence intervals. Heterogeneity was determined by Cochran’s Q and Higgins’ I2 tests, while publication bias was assessed using funnel plots and Egger’s test. Meta-analysis was performed using the Comprehensive Meta-Analysis software version 3 (Biostat, Englewood, NJ, USA, 2004). RNA-seq data (processed with FastQC and Trimmomatic v0.40) were pseudo-aligned using Salmon v1.10.0^37^ with an index based on the reference genome GRCh38.p14. Raw count data were analyzed with tximport v1.26.1 and ensembldb v3.17 packages using R v4.2.2. Differential gene expression analysis was conducted using the limma-voom method^38^. PAM50 classification of BC cell lines, was carried out employing the algorithm provided by the genefu package^39^. After assessing the normality of the data (Shapiro–Wilk test), Tukey’s multiple comparisons test was used to determine differences in qRT-PCR mean gene expression values between the studied groups. P-values < 0.05 were considered significant. Analyses were carried out using GraphPad Prism v.7.0a statistical software and R: The R Project for Statistical Computing v4.2.2. (http://www.r-project.org/).

## Results

The methodology employed for the process of searching microarray database repositories resulted in the identification of approximately 116,000 datasets. After applying the inclusion criteria for eligibility assessment, 58 datasets of tumor samples corresponding to 8,798 patients with BC were selected (Supplementary table 1). Table 1 describes the clinicopathological characteristics and the number of cases analyzed in each dataset for the meta-analysis.

**Table 1:** Clinicopathological characteristics of BC patients included in meta-analysis.

Characteristics		n (%)
Stage	I	564 (32.4)
	II	796 (45.7)
	III	355 (20.4)
	IV	27 (1.5)
Grade	1	817 (13.8)
	2	2373 (40.0)
	3	2739 (46.2)
Tumor size (T)	T1	719 (34.1)
	T2	923 (43.8)
	T3	240 (11.4)
	T4	227 (10.8)
Lymph node status (N)	N0	2665 (58.8)
	N1	1531 (33.8)
	N2	189 (4.2)
	N3	145 (3.2)
Metastases (M)	M0	994 (80.4)
	M1	242 (19.6)
HER2	Negative	4020 (77.5)
	Positive	1165 (22.5)
IHC—Surrogate subtypes	Luminal A	1505 (55.9)
	Luminal B	374 (13.9)
	HER2-enriched	216 (8.0)
	TNBC	599 (22.2)
Intrinsic molecular subtypes—PAM50	Luminal A	1258 (38.9)
	Luminal B	706 (21.9)
	HER2-enriched	396 (12.3)
	Basal-like	610 (18.9)
	Normal-like	261 (8.1)

HER2, Human Epidermal Growth Factor Receptor 2; IHC, immunohistochemistry; TNBC, Triple negative breast cancer.

### *High AR/ESR1 ratio is associated with HER2* + *cases*

In the meta-analysis, thirty-nine microarray datasets with clinical information from patients with BC related to HER2 status were included. The results indicate that HER2-positive (HER2 +) patients are significantly associated with *AR/ESR1* ≥ 2.0 (Odds ratio: 2.731; 95% CI: 1.621–4.6; p < 0.001) (Supplementary Fig. 1). The heterogeneity of this analysis was moderate, and no evidence of publication bias was observed, according to the funnel plot and Egger’s test (Supplementary Fig. 2). No statistical evidence was found associating high *AR/ESR1* ratio values (> 2.0) with other clinical characteristics.

### *High AR/ESR1 ratio is associated withLuminal B and HER2-enriched IHC—surrogate subtypes*

**Fig. 1:**
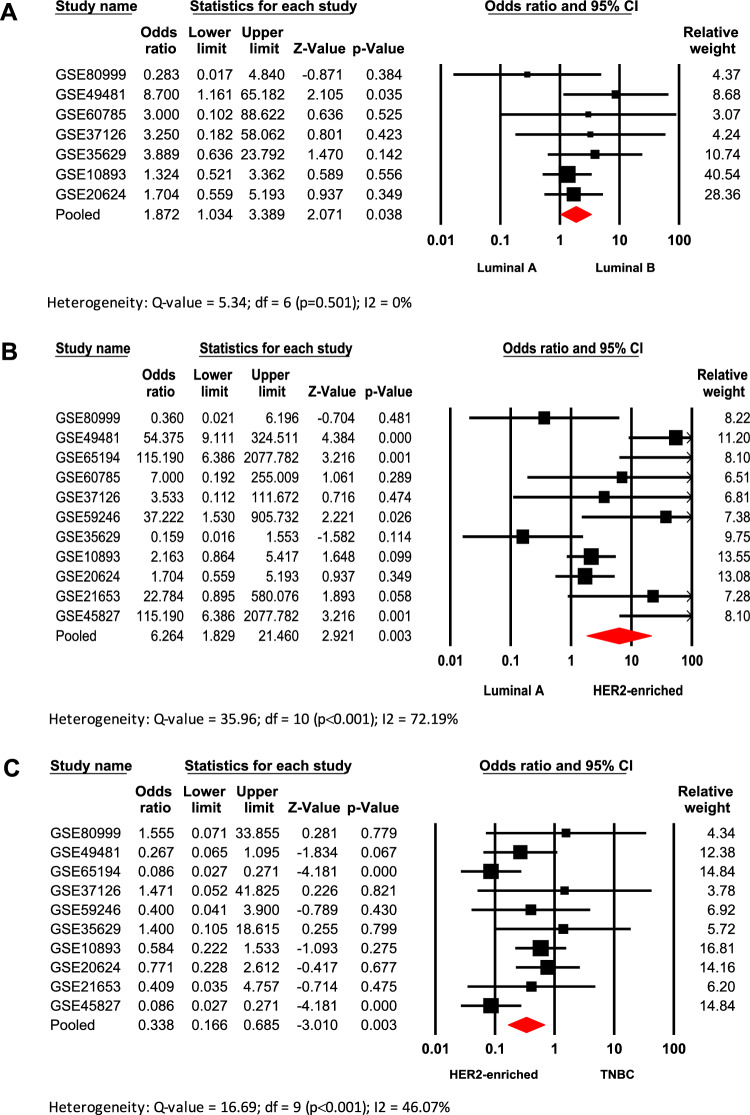
Association of high *AR/ESR1* ratio values with IHC-surrogate subtypes of worse prognosis (Luminal B and HER2-enriched). Forest plots compare Luminal A vs. Luminal B (**A**), Luminal A vs. HER2-enriched (**B**), and HER2-enriched vs. TNBC (C) IHC-surrogate subtypes. Odds ratios (ORs) for each dataset are shown as black squares, whose size reflects the relative weight of each study; horizontal lines indicate 95% confidence intervals (CIs), and red diamonds represent the pooled OR with its 95% CI.

Given the relevance of the IHC classification system in the clinical setting, the association between *AR/ESR1* ratio expression and surrogate subtypes was analyzed. The meta-analysis included sixteen databases with clinical information from patients classified into the four surrogate subtypes as observed in Table 1. When comparing the *AR/ESR1* ratio across all surrogate subtypes (Supplementary table 2), it was observed that patients classified as Luminal B tumors are significantly associated with higher *AR/ESR1* ratio values (≥ 2.0) when compared to Luminal A tumors (Odds ratio: 1.872; 95% CI: 1.034–3.389; p = 0.038) (Fig. 1A). Similarly, tumors classified as HER2-enriched are significantly associated with *AR/ESR1* ≥ 2.0, compared to Luminal A tumors (Odds ratio: 6.264; 95% CI: 1.829–21.460; p = 0.003) (Fig. 1B) and TNBC (Odds ratio: 0.375; 95% CI: 0.183–0.768; p = 0.007) (Fig. 1C). In none of these cases was evidence of publication bias according to the funnel plots and Egger’s tests (Supplementary Fig. 3). However, the results indicate low heterogeneity in the Luminal B vs. Luminal A, moderate in the TNBC vs. HER2-enriched, and substantial in the HER2-enriched vs. Luminal A comparisons.

### *High AR/ESR1 ratio is associated with HER2-enriched intrinsic molecular subtype—PAM50*

**Fig. 2:**
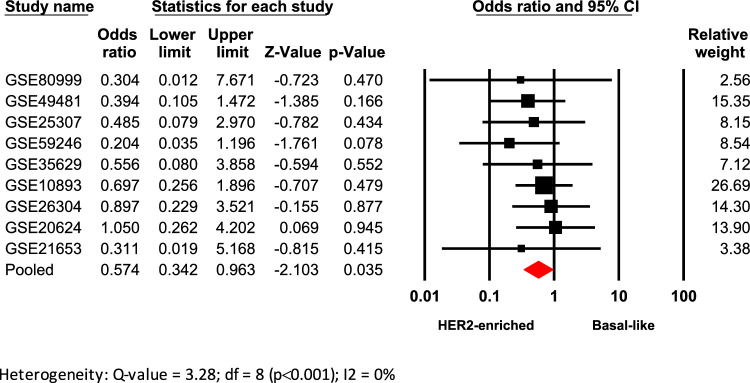
Association of high *AR/ESR1* ratio values with the HER2-enriched intrinsic molecular subtype. Forest plot compares HER2-enriched vs. Basal-like intrinsic molecular subtypes. Odds ratios (ORs) for each dataset are shown as black squares, whose size reflects the relative weight of each study; horizontal lines indicate 95% confidence intervals (CIs), and red diamonds represent the pooled OR with its 95% CI.

Given that the classification of BC subtypes in research studies is primarily based on gene expression profiles, clinical information from seventeen microarrays where BC patients were classified using the PAM50 algorithm, were included (Table 1). When comparing the *AR/ESR1* ratio across all molecular subtypes (Supplementary table 2), it was again observed that patients classified as HER2-enriched were significantly associated with *AR/ESR1* ratio values ≥ 2.0, compared to Basal-like tumors (Odds ratio: 0.574; 95% CI: 0.342–0.963; p = 0.035) (Fig. 2). Additionally, tumor tissues from patients classified as Normal-like were significantly associated with *AR/ESR1* ≥ 2.0, compared to Luminal A tumors (Odds ratio: 0.371; 95% CI: 0.145–0.949; p = 0.039) (Supplementary Fig. 4). Heterogeneity was low in the first case and moderate in the Normal-like vs. Luminal A comparison. No evidence of publication bias was observed in any of these analyses, according to the funnel plots and Egger’s tests (Supplementary Fig. 5).

### *High AR/PGR ratio is associated with worse clinicopathological characteristics*

In contrast to the *AR/ESR1* ratio, *AR/PGR* ratio values ≥ 1.54 were associated with high grades (G2-G3; Odds ratio: 1.353; 95% CI: 1.078–1.698; p = 0.009), larger tumor sizes (T2-T4; Odds ratio: 1.284; 95% CI: 1.015–1.626; p = 0.038), the presence of multiple positive lymph nodes (N2-N3; Odds ratio: 1.8; 95% CI: 1.331–2.436; p < 0.001), and also with HER2 positivity (HER2 +; Odds ratio: 2.084; 95% CI: 1.502–2.890; p < 0.001) (Supplementary Fig. 6 and 7). For the meta-analysis of each of the indicated characteristics, 38, 14, 33, and 38 microarray databases were included, respectively. In these analyses, only moderate heterogeneity was observed for HER2 + vs. HER2-. For the other comparisons, heterogeneity was low. Additionally, according to the funnel plots and Egger’s tests (Supplementary Fig. 8), no evidence of publication bias was observed in any case.

### *High AR/PGR ratio is associated withLuminalB and HER2-enriched IHC—surrogate subtypes*

**Fig. 3:**
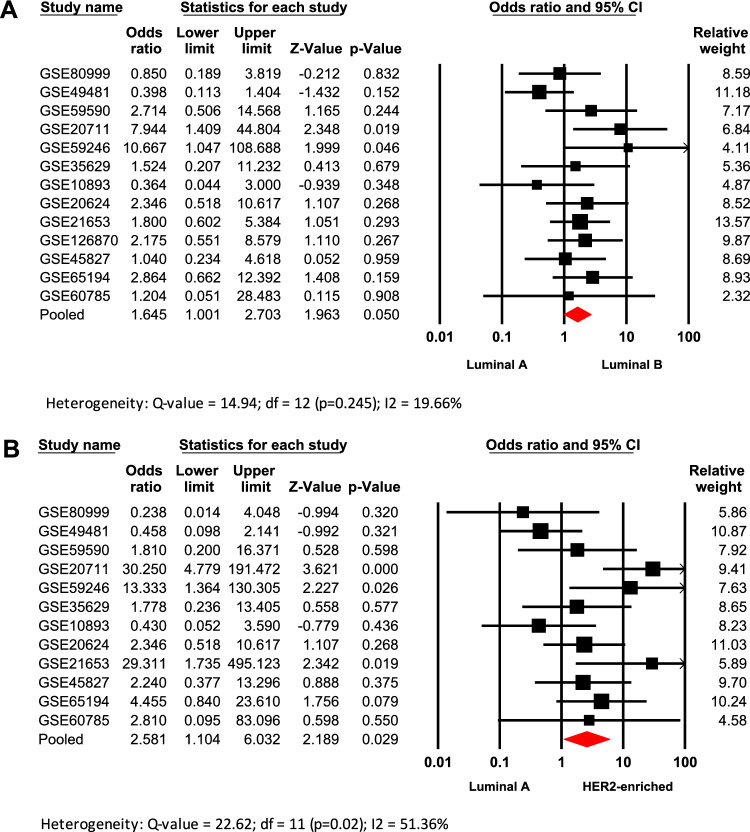
Association of high *AR/PGR* ratio values with IHC-surrogate subtypes of worse prognosis (Luminal B and HER2-enriched). Forest plots compare Luminal A vs. Luminal B (**A**) and Luminal A vs. HER2-enriched (**B**) IHC-surrogate subtypes. Odds ratios (ORs) for each dataset are shown as black squares, whose size reflects the relative weight of each study; horizontal lines indicate 95% confidence intervals (CIs), and red diamonds represent the pooled OR with its 95% CI.

For this analysis fifteen databases with clinical information from patients classified into the four surrogate subtypes, were included (Table 1). When comparing the *AR/PGR* ratio across all surrogate subtypes (Supplementary table 3), the results indicate that tumor tissues from patients classified as Luminal B (Odds ratio: 1.645; 95% CI: 1.001–2.703; p = 0.05) and HER2-enriched (Odds ratio: 2.581; 95% CI: 1.104–6.032; p = 0.029) are significantly associated with *AR/PGR* ≥ 1.54, compared to tissues from patients classified as Luminal A (Fig. 3). Heterogeneity in these analyses was low and substantial, respectively, and in both cases, no evidence of publication bias was observed according to the funnel plots and Egger’s tests (Supplementary Fig. 9).

### *High AR/PGR ratio is associated withLuminalB intrinsic molecular subtype—PAM50*

**Fig. 4:**
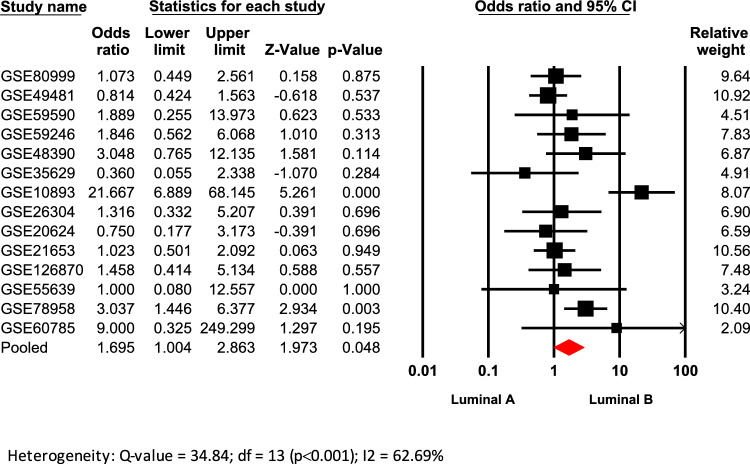
Association of high *AR/PGR* ratio values with the Luminal B intrinsic molecular subtype. Forest plot compares Luminal A vs. Luminal B intrinsic molecular subtypes. Odds ratios (ORs) for each dataset are shown as black squares, whose size reflects the relative weight of each study; horizontal lines indicate 95% confidence intervals (CIs), and red diamonds represent the pooled OR with its 95% CI.

For the meta-analysis clinical information from sixteen microarrays where BC patients were classified using the PAM50 algorithm, were included (Table 1). When comparing the *AR/PGR* ratio across all molecular subtypes (Supplementary table 3), it was observed that patients classified as Luminal B were significantly associated with *AR/PGR* ≥ 1.54, compared to Luminal A tumors (Odds ratio: 1.695; 95% CI: 1.004–2.863; p = 0.048) (Fig. 4). Moreover, tumor tissues from patients classified as Normal-like were significantly associated with *AR/PGR* ≥ 1.54, compared to Luminal A tumors (Odds ratio: 2.079; 95% CI: 1.304–3.316; p = 0.002) (Supplementary Fig. 10). Both analyses showed low heterogeneity and in none of these cases was evidence of publication bias according to the funnel plots and Egger’s tests (Supplementary Fig. 11).

### *mRNA gene expression of AR/ESR1 and AR/PGR ratios in BC cell lines*

**Fig. 5:**
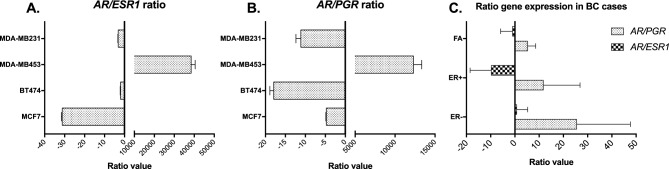
mRNA gene expression ratios determined by qRT-PCR. *AR/ESR1* ratio in BC cell lines (**A**). *AR/PGR* ratio in BC cell lines (**B**). Mean gene expression ratios in BC tissue of estrogen receptor-positive (ER +) patients, estrogen receptor-negative (ER-) patients and in fibroadenomes (FA) cases (**C**). Statistical differences (p < 0.05) were observed only for the *AR/PGR* ratio when ER- cases were compared with ER + and FA cases. p values: Tukey’s multiple comparisons test.

To further define the molecular subtype of the cell lines studied, the PAM50 classifier was used based on the RNA-seq data. The MCF7, BT474 and MDA-MB231 cell lines were classified as expected, Luminal A, Luminal B and basal-like, respectively. However, the MDA-MB453 cell line, representative of the apocrine/basal-like subtype, was classified as Luminal A. The qRT-PCR analysis showed that *ESR1* and *PGR* gene expressions were higher in cell lines representative of the Luminal subtype (MCF7 and BT474), while their levels were very low or absent in the MDA-MB453 and MDA-MB231 cell lines. Although *AR* gene expression was observed in the luminal cell lines, it was higher in BT474 with respect to MCF7. As expected, *AR* was expressed at much higher levels in MDA-MB453 cells than in the classical triple-negative cell line MDA-MB231, which had very low or absent mRNA levels of *AR*. The *ESR1*, *PGR*, and *AR* gene expression results were coincident with those observed by RNA-seq analysis (Supplementary Fig. 12). The qRT-PCR and RNA-seq analyses showed that, from the four cell lines studied, the only one with highly positive values for *AR/ESR1* and *AR/PGR* ratios was MDA-MB453, representative of triple negative apocrine tumors but classified by PAM50 as Luminal A. In agreement with the expression levels observed for each gene, MCF7 was the cell line with the lowest *AR/ESR1* ratio, while BT474 had the lowest *AR/PGR* ratio levels (Fig. 5A and B).

### *mRNA gene expression of AR/ESR1 and AR/PGR ratios in tumor tissues*

Receptor expression analysis by immunohistochemistry showed that two of six tissues from BC patients (TT-44 and TT-217) were reported as ER- (ER-/PgR-/HER2+), while the remaining four were classified as ER+ (ER+/PgR +/HER2-) (Supplementary Fig. 13). Accordingly, ER- cases showed low *ESR1* and *PGR* gene expression levels with respect to ER+ cases. Likewise, the expression level of the *AR* gene was clearly higher in ER+ cases. Gene expression analysis in FA showed higher mRNA levels for hormone receptors like those observed in ER+ tumors (Supplementary Fig. 14). The average FC value of the *AR/ESR1* ratio of the four ER+ cases showed negative values (−9.72), while for the ER- cases it was positive (0.72). In contrast, the *AR/PGR* ratio was clearly positive in both ER+ and ER- cases. Tissue from FA showed the same behavior for both ratios (*AR/ESR1 & AR/PGR*) as observed in ER+ BCs (Fig. 5C).

## Discussion

We conducted multiple analyses on 58 transcriptomic data sets of gene microarrays, where it was observed that high levels of *AR* gene expression relative to *ESR1* and *PGR* (≥ 2.0 and ≥ 1.54, respectively) may be associated with BC patients having poorer clinical features, as well as Luminal B, and HER2-enriched subtypes. Furthermore, positive values of both ratios were also observed in the ER- cell line MDA-MB453 and in tissue from ER- BC patients. Together our findings suggest that higher or positive *AR/ESR1* and *AR/PGR* ratio values may be associated with BC cases presenting more aggressive clinical and biological features, therefore with worse prognosis.

Previous studies have reported associations between AR/ER ≥ 2.0 with poorer clinical features like positive lymph nodes, larger tumor sizes, higher histological grades and overexpression/amplification of the HER2 gene in ER+ BC patient^20,21,40^. Independently of the ER status our meta-analysis confirmed the relationship among HER2+ BC patients and higher *AR/ESR1* ratio values (≥ 2.0). Interestingly, the analyses also revealed associations between BC patients with *AR/PGR* ≥ 1.54 and higher histological grades, larger tumor sizes, positive lymph nodes and HER2 positivity (Supplementary Fig. 6 and 7), which to the best of our knowledge, have not been previously reported. Only the study of Amine et al. recently reported a correlation between non-metastatic ER+/HER2- BC patients with AR/PgR > 1.63 and early stages of the disease^41^. These contradictory results may be due to the limited and specific cohort (non-metastatic HR+/HER2-) used in the cited study. Our data helps explain why higher *AR/ESR1* and *AR/PGR* ratios have previously been linked to shorter survival, suggesting their potential as markers of aggressiveness and poor prognosis in BC^20,22,42,43^.

BC has been classified into intrinsic molecular subtypes based on profiling the mRNA expression of at least 50 genes (PAM50)^4^. However, in clinical practice, subtype stratification is determined by assessing the IHC expression of ER, PgR, HER2, and the proliferation marker Ki-67 (IHC surrogate classification). Both systems have improved BC tumor classification for better prognosis and treatment, though some discrepancies have been reported when comparing their subtype definitions^44^. Here when comparing luminal tumors (ER+), the association between BC subtypes (both IHC-surrogate and molecular) and ratio values showed that tissues from BC patients with *AR/PGR* ≥ 1.54 have a significant trend to be classified as Luminal B when compared to Luminal A tumors (Figs. 3A and 4). A similar pattern was observed for patients with *AR/ESR1* ≥ 2.0 for both classification systems (Fig. 1A; Supplementary table 2). These results are consistent with previous findings indicating a strong correlation between AR expression and the co-expression of ER and PgR in BC^45^. They are also in agreement with reports suggesting that cases with higher *AR/ESR1* ratios are more likely to be classified as Luminal B tumors^21^. Elevated ratios may indicate a preferential activation of AR-driven transcriptional programs linked to more aggressive features, such as those observed in Luminal B tumors^46^. In fact, some reports have shown elevated expression of gene proliferation markers in Luminal B cases with higher *AR/ESR1* ratio levels^40^, while others implicate AR as an oncogenic factor that induces Epithelial-Mesenchymal Transition (EMT)^47^ or activates the JAK/STAT pathway to promote plasticity toward an aggressive, ER+ resistant subtype^29^. Although AR agonists may counteract ER oncogenic effects in luminal tumors^48^, studies have shown that AR overexpression in ER+ models can lead to hormone therapy resistance^49,50^. Our results suggest that assessing *AR/ESR1* expression ratios could help predict tumor response and support the use of AR antagonists in cases with elevated ratios.

Our analyses showed that higher *AR/ESR1* ratio values are predominantly associated with HER2-enriched tumors (Supplementary table 2), particularly when compared to Luminal A, TNBC (IHC-surrogate. Figure 1B and C), and basal-like cases (molecular subtype. Figure 2). This result, in addition to confirming previous findings^21,40^, aligns with the observed association between HER2+ cases and higher *AR/ESR1* ratios (Supplementary Fig. 2). These findings reinforce the cooperative interaction between AR and HER2^51–53^, which enhances their signaling and contributes to the aggressiveness and poor prognosis of HER2-enriched BCs^40,54,55^. Accordingly, they support ongoing research into the efficacy of combined therapies targeting both pathways, such as enzalutamide (AR) and trastuzumab (HER2)^56^. Contrary to *AR/ESR1* ratio, higher *AR/PGR* ratio values (≥ 1.54) did not show a clear trend to be associated with the HER2-enriched subtype. The association was observed only when HER2-enriched was compared with Luminal A IHC-surrogate subtype (Supplementary table 3). Although several studies have established functional relationships between AR and ER proteins^17,45,57,58^, there are very few studies attempting to establish such functional relationships between PgR with AR and even with HER2. It has been reported that AR, like PgR, can recruit Src and activate MAPK and PI3K/AKT pathways^59^, key signaling routes also triggered by HER2, suggesting a potential cross-talk among AR, HER2, and PgR that may drive gene dysregulation and promote proliferation, migration, and invasiveness in BC cells.

The meta-analysis also revealed that Normal-like cases are more likely to have higher expression levels of *AR/ESR1* (≥ 2.0) and *AR/PGR* (≥ 1.54) when compared to Luminal A tumors (Supplementary Figs. 4 and 10). Although it is interesting, this information must be interpreted with caution. The clinical relevance of the Normal-like subtype remains in doubt, since it still grouped with normal tissue samples and fibroadenomas^60,61^. Although classified as less aggressive, some studies have shown that Normal-like BCs can overexpress basal epithelial genes (*CK5, CK14, CK17, laminin*, etc.)^61–63^, suggesting similarities with more aggressive subtypes such as basal-like and Claudin-low tumors^64–67^. In this context, our findings support the notion that Normal-like cases with *AR/ESR1* ≥ 2.0 and/or *AR/PGR* ≥ 1.54 may share unfavorable biological traits and prognosis closer to aggressive subtypes than to luminal tumors.

Regarding cell lines analysis, both the *AR/ESR1* and *AR/PGR* ratios showed low (negative) values in ER+ cells (MCF7 and BT474), however the *AR/ESR1* ratio in BT474 was higher compared to MCF7. (Fig. 5A and B). These results are consistent with the meta-analysis findings, since BT474 is commonly used as a Luminal B model and also shows elevated HER2 expression^68^. The negative ratio values observed in the ER- MDA-MB231 cells (Fig. 5A and B), attributed to low or absent expression of all hormone receptors (Supplementary Fig. 12D), may reflect most of basal-like or TNBC cases in the meta-analysis, which also exhibited low values for both ratios. In contrast, the highly positive values observed for both ratios in MDA-MB453 cells (ER-. Figure 5A and B), could correspond to Normal-like cases within the meta-analysis. As previously mentioned, these cases exhibit variable characteristics that challenge its classification, as the high AR levels observed in the MDA-MB453 cell line, which has led to its recategorization as Luminal A. For the *AR/ESR1* ratio, the differences observed in cell lines were also evident in the small group of tumor tissues studied (Fig. 5C). Thus, its positive or higher values tend to be associated with characteristics of worse prognosis, as previously established^20,21^. Similar to MDA-MB453 cells, the *AR/PGR* ratio values were highly positive in both ER+ and ER- BC cases, but contrary to results reported by other authors^42,43^.

It is important to highlight that our meta-analysis had limitations, primarily due to variations in sample processing methodologies, the different types of microarrays used, discrepancies in the number of patients analyzed across studies, and the lack of clear standardization of optimal cut-off values for both ratios. To address these limitations, future studies will need to include larger patient cohorts evaluated using more sensitive and specific methodologies (*e.g.,* RNA-seq) along with comprehensive clinicopathological information and appropriate follow-up to enable analysis of the relationship between *AR/ESR1* and *AR/PGR* ratios and survival parameters as well as BC progression. Prospective studies that include representative numbers of tumor tissues from different BC subtypes will be highly useful in better defining the potential differentiating role of these ratios in BC, particularly the *AR/PGR* ratio.

## Conclusions

Our results confirm that higher *AR/ESR1* and *AR/PGR* ratio values are mainly associated with HER2-Enriched and Luminal B BC subtypes, both linked to poorer prognosis. Analyses of mRNA expression in cell lines and tumor tissues further support the utility of these ratios in identifying tumors with non-luminal characteristics (i.e., ER−). These findings highlight the clinical relevance of evaluating ER, PgR, and AR markers jointly—not only at the protein but also at the transcript level—to better stratify patients. This approach could serve as an additional tool for identifying BC cases that may benefit from targeted therapies against specific hormone receptors, such as AR, thus contributing to more personalized and effective treatment strategies.

## Supplementary Information

Supplementary Information 1.

Supplementary Information 2.

Supplementary Information 3.

## Data Availability

Sequence data supporting the findings of this study have been deposited in the NCBI Bioprojects Repository. Accession Number: PRJNA1089410. https://www.ncbi.nlm.nih.gov/bioproject/1,089,410 More information about the datasets or materials used and/or analyzed during the current study are available from the corresponding authors on reasonable request.
