# Supplementary material for: mRNA ratios of AR to ESR1 and PGR distinguish breast cancer subtypes based on public datasets and experimental models
Source: Sci Rep. 2025 Jul 1;15:21793. doi: 10.1038/s41598-025-06856-3 (PMC12216603; doi:10.1038/s41598-025-06856-3)
Supplement: Supplementary file 2 — Supplementary Information 2. [file 41598_2025_6856_MOESM2_ESM.pdf]

Study name	Statistics for each study					Relative weight
	Odds ratio	Lower limit	Upper limit	Z-Value	p-Value	
GSE80999	0.178	0.011	2.998	-1.199	0.231	2.50
GSE49481	6.563	2.210	19.486	3.388	0.001	6.67
GSE65194	5.333	1.973	14.413	3.300	0.001	7.01
GSE60785	3.222	0.114	91.341	0.686	0.493	1.93
GSE37126	1.133	0.106	12.129	0.103	0.918	3.20
GSE31364	0.306	0.088	1.063	-1.864	0.062	6.12
GSE20194	11.256	0.453	279.922	1.477	0.140	2.06
GSE59246	6.400	1.065	38.473	2.028	0.043	4.45
GSE71258	10.412	1.016	106.647	1.974	0.048	3.28
GSE29044	0.939	0.056	15.679	-0.044	0.965	2.51
GSE43365	64.333	3.106	1332.673	2.693	0.007	2.25
GSE35629	2.111	0.598	7.448	1.162	0.245	6.07
GSE10893	1.552	0.786	3.064	1.266	0.206	8.11
GSE16987	0.515	0.111	2.379	-0.850	0.395	5.20
GSE26639	7.836	1.622	37.849	2.562	0.010	5.06
GSE26304	2.652	0.562	12.511	1.232	0.218	5.13
GSE20271	9.529	3.246	27.973	4.103	0.000	6.71
GSE20624	1.732	0.752	3.989	1.291	0.197	7.58
GSE21653	3.821	0.336	43.518	1.080	0.280	3.09
GSE17907	0.415	0.015	11.828	-0.514	0.607	1.93
GSE19615	14.808	3.044	72.030	3.339	0.001	5.04
GSE4056	0.346	0.017	7.114	-0.688	0.492	2.26
GSE19697	2.867	0.090	91.045	0.597	0.551	1.83
Pooled	2.731	1.621	4.600	3.775	0.000	

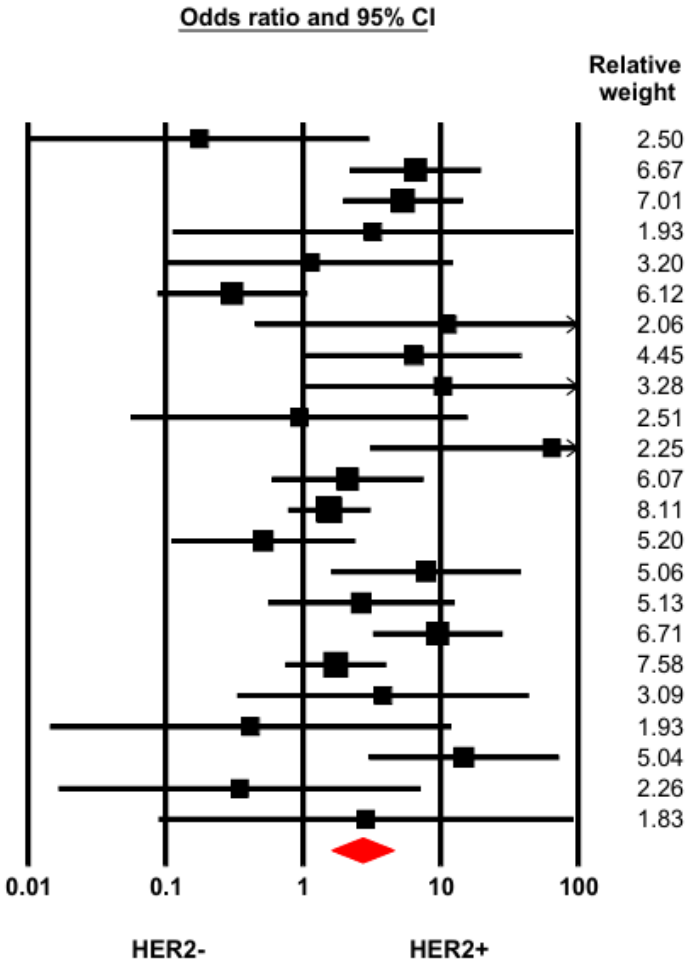

Heterogeneity: Q-value = 5.34; df = 6 (p = 0.501); I^2 = 0%

Supplementary Figure 1. High *AR/ESR1* ratio is associated with HER2+ cases. Forest plot of Odds ratio comparing *AR/ESR1* levels in HER2- BC patients vs. HER2+ BC patients. Odds ratio for each dataset are represented by the squares, and horizontal line crossing the square represents the 95% CI. The red diamonds represent the estimated overall effect.

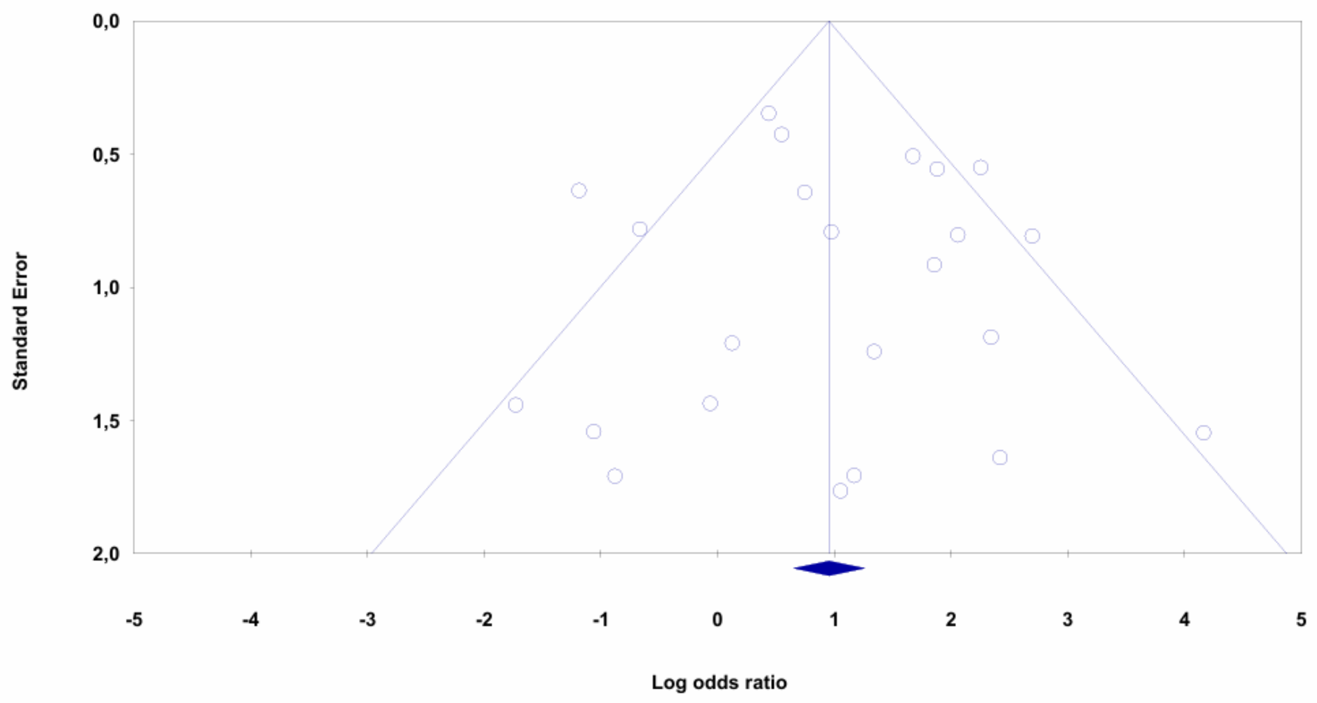

Egger's test: 0.16; 95%CI: -1.30 to 1.62; p = 0.824

Supplementary Figure 2. Funnel plot for main meta-analyses. Association of high *AR/ESR1* ratio values in HER2- BC patients vs. HER2+ BC patients.

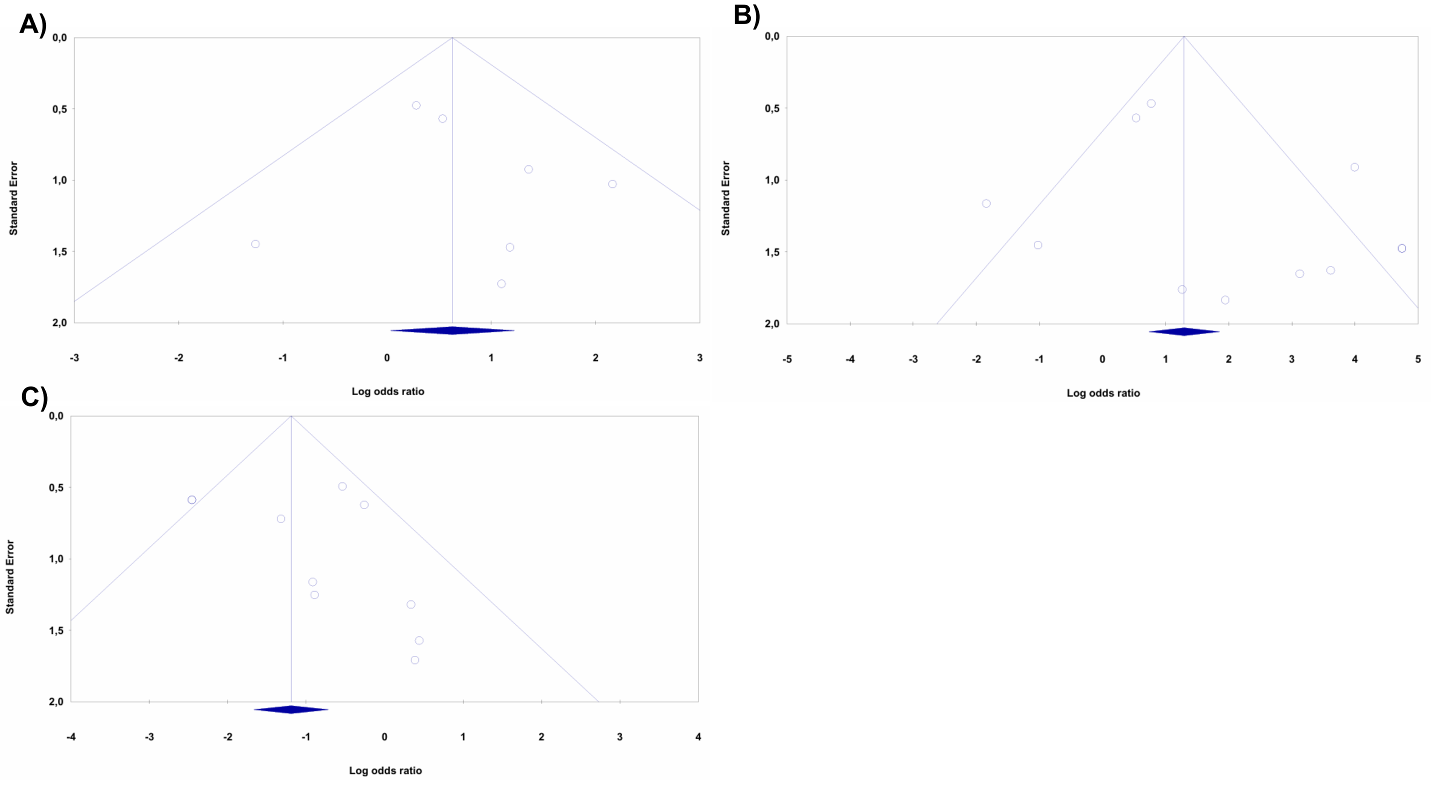

Supplementary Figure 3. Funnel plots for main meta-analyses. Association between *AR/ESR1* levels in BC patients classified with IHC-surrogate subtypes Luminal A vs. Luminal B **(A)** / HER2-enriched **(B)** and HER2-enriched vs. TNBC **(C).**

Study name	Odds ratio	Lower limit	Upper limit	Z-Value	p-Value	Relative weight
GSE80999	1.571	0.335	7.368	0.573	0.566	18.46
GSE49481	0.005	0.000	0.126	-3.249	0.001	7.18
GSE60785	1.000	0.034	29.187	0.000	1.000	6.42
GSE25307	0.401	0.088	1.838	-1.176	0.239	18.73
GSE35629	0.400	0.020	8.071	-0.598	0.550	7.74
GSE10893	0.300	0.112	0.804	-2.395	0.017	26.20
GSE26304	0.783	0.046	13.390	-0.169	0.866	8.45
GSE20624	0.164	0.006	4.253	-1.088	0.277	6.81
Pooled	0.371	0.145	0.949	-2.068	0.039	

(Header row: "Statistics for each study" spans Odds ratio through p-Value.)

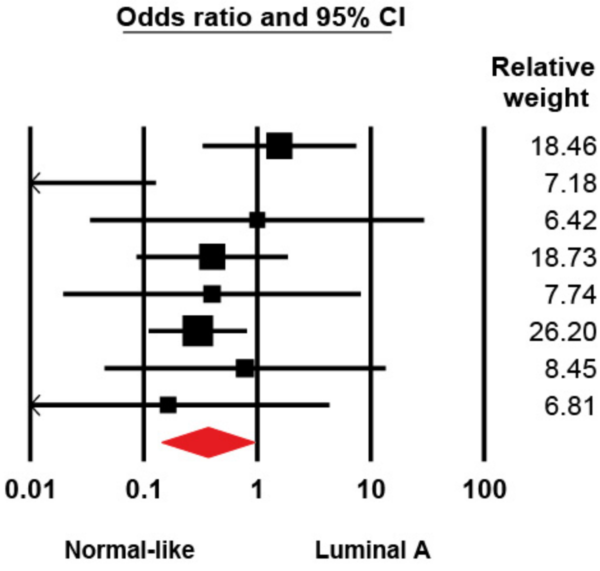

Heterogeneity: Q-value = 11.29; df = 7 (p = 0.126); I2 =38%

Supplementary Figure 4. High *AR/ESR1* ratio is associated with Normal-like intrinsic molecular subtype - PAM50. Forest plot of Odds ratio comparing *AR/ESR1* levels in BC patients classified as Normal-like vs. Luminal A. Odds ratio for each dataset are represented by the squares, and horizontal line crossing the square represents the 95% CI. The red diamonds represent the estimated overall effect.

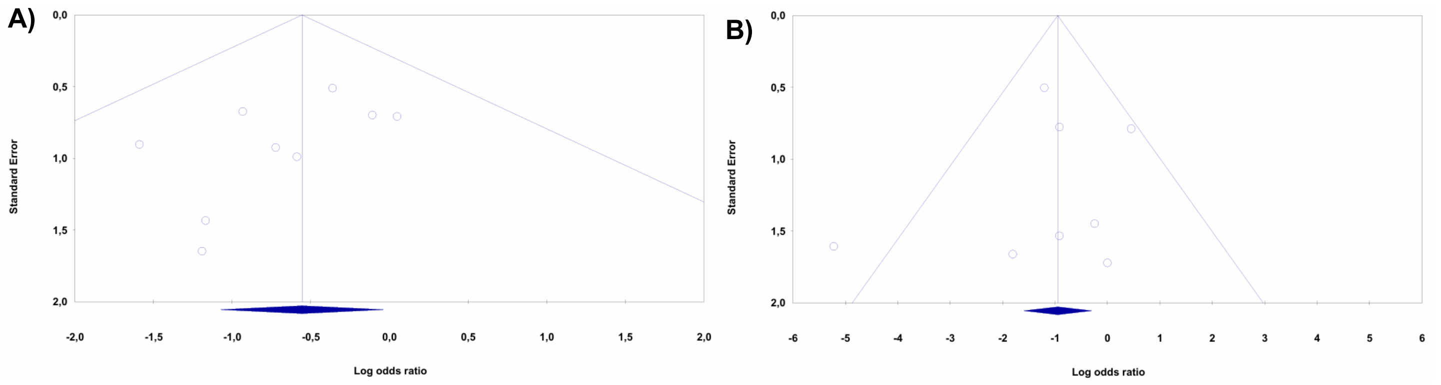

Egger's test: -0.97; 95%CI: -2.45 to 0.51; p = 0.164

Egger's test: -0.51; 95%CI: -3.14 to 2.11; p = 0.65

Supplementary Figure 5. Funnel plots for main meta-analyses. Association between *AR/ESR1* levels in BC patients classified with intrinsic molecular subtypes HER2-enriched vs. Basal-like **(A)** and Normal-like vs. Luminal A **(B)**.

A

Study name	Odds ratio	Lower limit	Upper limit	Z-Value	p-Value	Relative weight
GSE49481	0.865	0.418	1.790	-0.390	0.697	8.07
GSE60785	2.552	0.126	51.740	0.610	0.542	0.56
GSE42568	0.889	0.254	3.117	-0.184	0.854	3.06
GSE31863	0.565	0.185	1.730	-0.999	0.318	3.78
GSE24185	0.684	0.080	5.855	-0.347	0.729	1.09
GSE22219	0.264	0.036	1.931	-1.313	0.189	1.27
GSE20194	1.603	0.524	4.905	0.827	0.408	3.79
GSE10810	0.676	0.025	18.525	-0.232	0.816	0.47
GSE8757	1.077	0.507	2.286	0.193	0.847	7.61
GSE7390	1.832	0.792	4.238	1.414	0.157	6.33
GSE86166	0.950	0.457	1.972	-0.139	0.890	7.99
GSE59246	1.720	0.519	5.696	0.888	0.375	3.34
GSE61304	13.245	0.697	251.596	1.720	0.085	0.59
GSE29044	1.353	0.115	15.901	0.240	0.810	0.83
GSE43365	1.083	0.277	4.242	0.115	0.909	2.61
GSE45255	3.200	1.060	9.658	2.064	0.039	3.87
GSE10893	2.111	0.257	17.329	0.696	0.487	1.14
GSE16987	1.653	0.202	13.545	0.468	0.640	1.14
GSE26639	2.095	0.730	6.013	1.375	0.169	4.22
GSE26304	0.393	0.020	7.856	-0.611	0.541	0.57
GSE20271	9.429	0.559	159.137	1.556	0.120	0.64
GSE20624	0.441	0.084	2.330	-0.964	0.335	1.79
GSE21653	1.751	0.898	3.414	1.646	0.100	9.26
GSE1561	1.603	0.077	33.457	0.304	0.761	0.55
GSE17907	10.000	0.520	192.254	1.527	0.127	0.58
GSE19615	0.243	0.013	4.403	-0.958	0.338	0.61
GSE55639	0.353	0.029	4.350	-0.813	0.416	0.80
GSE78958	1.758	0.926	3.337	1.725	0.085	9.88
GSE11121	0.640	0.230	1.783	-0.854	0.393	4.44
GSE4056	0.480	0.028	8.348	-0.504	0.614	0.62
GSE80999	2.419	0.835	7.006	1.628	0.104	4.15
GSE31364	1.759	0.418	7.402	0.770	0.441	2.37
GSE20711	9.232	1.906	44.715	2.761	0.006	1.98
Pooled	1.353	1.078	1.698	2.609	0.009	

Statistics for each study

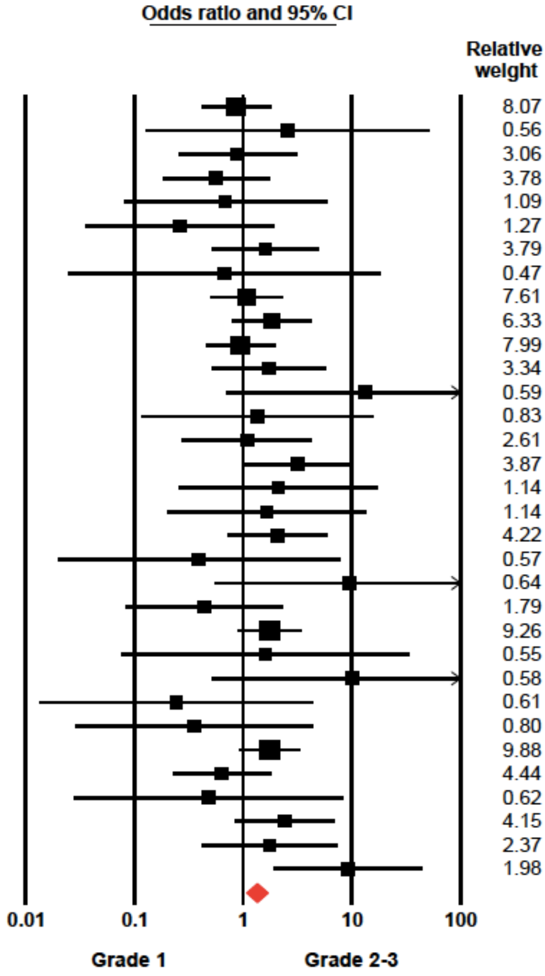

Heterogeneity: Q-value = 34.32; df = 32 (p=0.357); I2 = 6.75%

B

Study name	Odds ratio	Lower limit	Upper limit	Z-Value	p-Value	Relative weight
GSE89116	3.000	0.088	102.055	0.611	0.542	0.73
GSE20194	1.838	0.961	3.516	1.840	0.066	21.74
GSE10810	1.667	0.056	49.947	0.294	0.768	0.79
GSE61304	1.833	0.432	7.774	0.822	0.411	4.38
GSE43365	3.242	0.178	59.161	0.794	0.427	1.08
GSE10893	1.346	0.052	34.900	0.179	0.858	0.86
GSE20271	1.614	0.142	18.365	0.386	0.700	1.55
GSE10510	1.173	0.320	4.302	0.241	0.809	5.42
GSE1561	0.527	0.022	12.820	-0.393	0.694	0.90
GSE102484	1.870	1.276	2.740	3.208	0.001	62.55
Pooled	1.800	1.331	2.436	3.812	0.000	

Statistics for each study

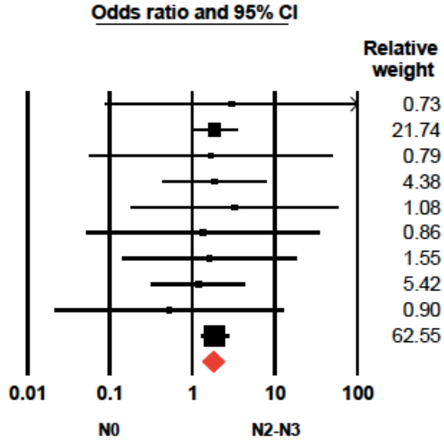

Heterogeneity: Q-value = 1.31; df = 32 (p=0.998); I2 = 0%

Supplementary Figure 6. High *AR/PGR* ratio value is associated with BC patients having Grade 1 vs. Grade 2-3 **(A)** and with BC patients having N0 vs. N2-N3 **(B)**. Odds ratio for each dataset are represented by the squares, and horizontal line crossing the square represents the 95% CI. The red diamonds represent the estimated overall effect.

A

Study name	Odds ratio	Lower limit	Upper limit	Z-Value	p-Value	Relative weight
GSE89116	2.760	0.102	74.777	0.603	0.546	0.51
GSE20194	0.832	0.347	1.993	-0.414	0.679	7.28
GSE10810	2.692	0.118	61.441	0.621	0.535	0.57
GSE61304	0.952	0.263	3.448	-0.074	0.941	3.36
GSE20271	2.006	0.098	41.232	0.451	0.652	0.61
GSE21653	1.142	0.610	2.138	0.414	0.679	14.12
GSE10510	1.295	0.533	3.146	0.571	0.568	7.06
GSE1561	0.649	0.060	7.070	-0.355	0.722	0.97
GSE17907	1.333	0.117	15.199	0.232	0.817	0.94
GSE102484	1.380	1.015	1.875	2.056	0.040	59.07
GSE19697	2.867	0.090	91.045	0.597	0.551	0.46
GSE31364	1.477	0.518	4.212	0.729	0.466	5.06
Pooled	1.284	1.015	1.626	2.080	0.038	

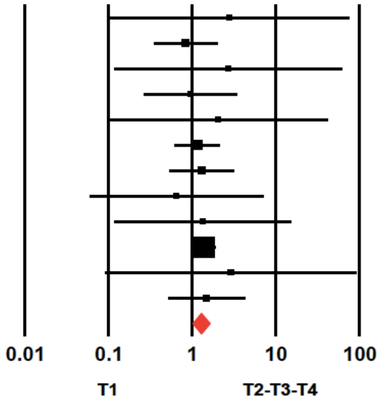

Heterogeneity: Q-value = 2.6; df = 11 (p=0.995); I2 = 0%

B

Study name	Odds ratio	Lower limit	Upper limit	Z-Value	p-Value	Relative weight
GSE89116	1.667	0.283	9.822	0.564	0.572	2.26
GSE49481	0.482	0.178	1.303	-1.438	0.150	4.16
GSE59590	1.818	0.468	7.059	0.864	0.388	3.12
GSE65194	17.519	6.227	49.281	5.426	0.000	4.03
GSE60785	0.365	0.018	7.350	-0.658	0.511	1.01
GSE41998	4.700	1.842	11.990	3.239	0.001	4.34
GSE24185	3.947	0.211	73.905	0.919	0.358	1.05
GSE20194	1.950	1.054	3.605	2.129	0.033	5.45
GSE129559	2.225	0.865	5.727	1.658	0.097	4.31
GSE86166	3.574	1.674	7.633	3.291	0.001	4.95
GSE59246	4.568	1.418	14.719	2.545	0.011	3.62
GSE71258	4.265	0.515	35.341	1.344	0.179	1.77
GSE29044	1.753	0.573	5.362	0.984	0.325	3.77
GSE43365	5.135	0.290	90.969	1.116	0.265	1.09
GSE45255	1.941	0.966	3.898	1.864	0.062	5.16
GSE35629	1.533	0.346	6.794	0.563	0.574	2.81
GSE10893	0.392	0.086	1.790	-1.209	0.227	2.75
GSE16987	0.637	0.136	2.990	-0.572	0.567	2.69
GSE22597	0.977	0.377	2.536	-0.048	0.962	4.29
GSE26639	5.671	2.833	11.353	4.900	0.000	5.17
GSE26304	1.605	0.620	4.157	0.975	0.330	4.29
GSE20271	1.241	0.062	24.720	0.141	0.888	1.02
GSE20624	3.360	0.995	11.341	1.953	0.051	3.49
GSE21653	2.997	1.004	8.942	1.968	0.049	3.85
GSE17907	2.417	0.200	29.234	0.694	0.488	1.37
GSE19615	7.552	0.420	135.910	1.371	0.170	1.08
GSE126870	2.175	0.551	8.579	1.110	0.267	3.08
GSE4056	1.150	0.267	4.959	0.187	0.851	2.87
GSE19697	0.610	0.022	16.666	-0.293	0.769	0.85
GSE80999	0.404	0.120	1.361	-1.463	0.144	3.50
GSE31364	0.806	0.214	3.035	-0.319	0.749	3.20
GSE20711	7.130	2.196	23.149	3.269	0.001	3.60
Pooled	2.084	1.502	2.890	4.400	0.000	

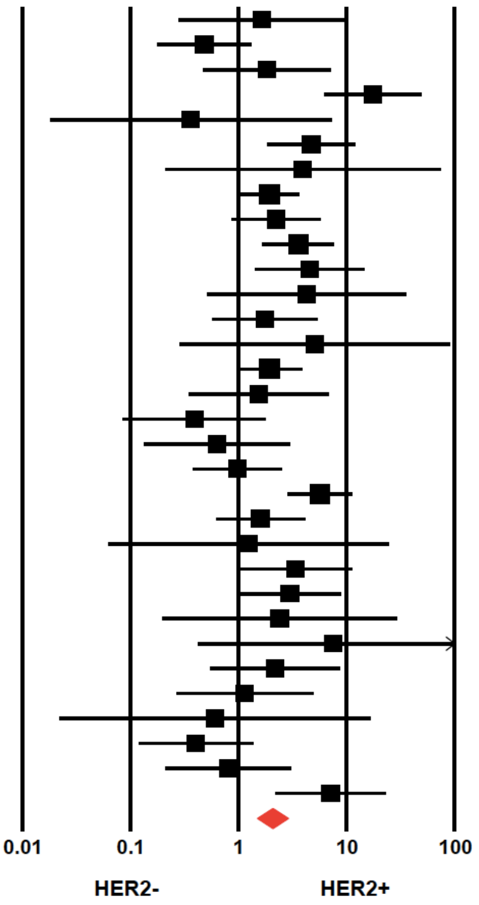

Heterogeneity: Q-value = 67.28; df = 31 (p<0.001); I2 = 53.93%%

Supplementary Figure 7. High *AR/PGR* ratio value is associated with BC patients having T1 vs. T2-T3-T4 **(A)** and with BC patients having HER2- vs. HER2+**(B)**. Odds ratio for each dataset are represented by the squares, and horizontal line crossing the square represents the 95% CI. The red diamonds represent the estimated overall effect.

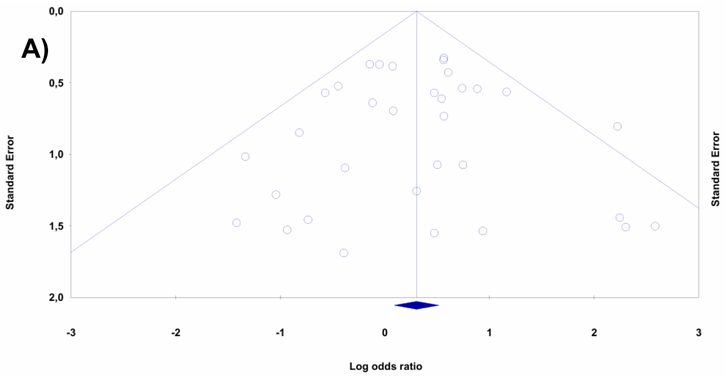

Egger’s test: 0.003; 95%CI: -0.79 to 0.80; p = 0.993

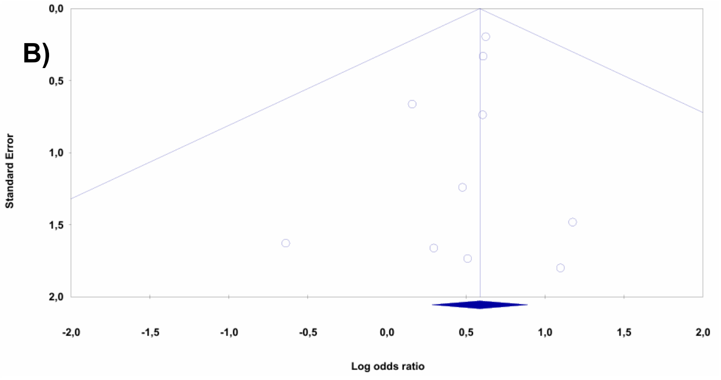

Egger’s test: -0.16; 95%CI: -0.56 to 0.25; p = 0.4

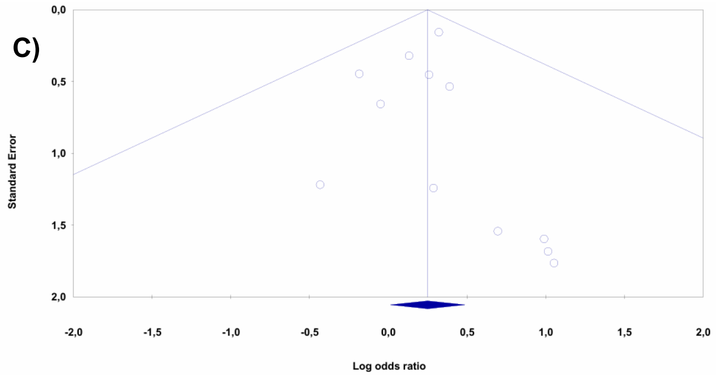

Egger’s test: 0.01; 95%CI: -0.48 to 0.50; p = 0.952

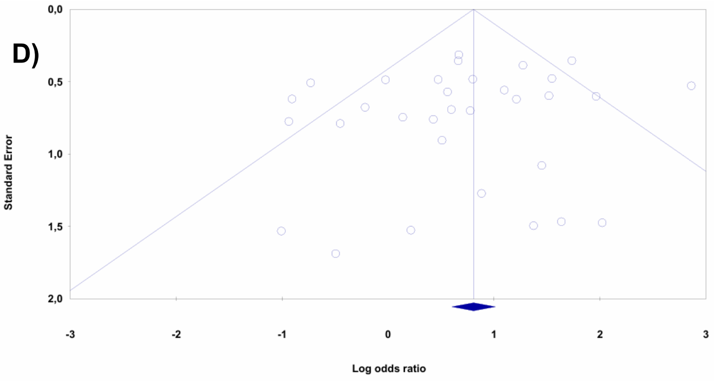

Egger’s test: -0.65; 95%CI: -1.98 to 0.68; p = 0.328

Supplementary Figure 8. Funnel plots for main meta-analyses. Association of high *AR/PGR* ratio values in BC patients with histological grade 1 vs. histological grades 2-3 **(A)**, negative lymph nodes (N0) vs. multiple positive lymph nodes (N1-N2) **(B)**, T1 tumoral size vs T2-T3-T4 tumoral sizes **(C)**, and HER2- BC patients vs. HER2+ BC patients **(D)**.

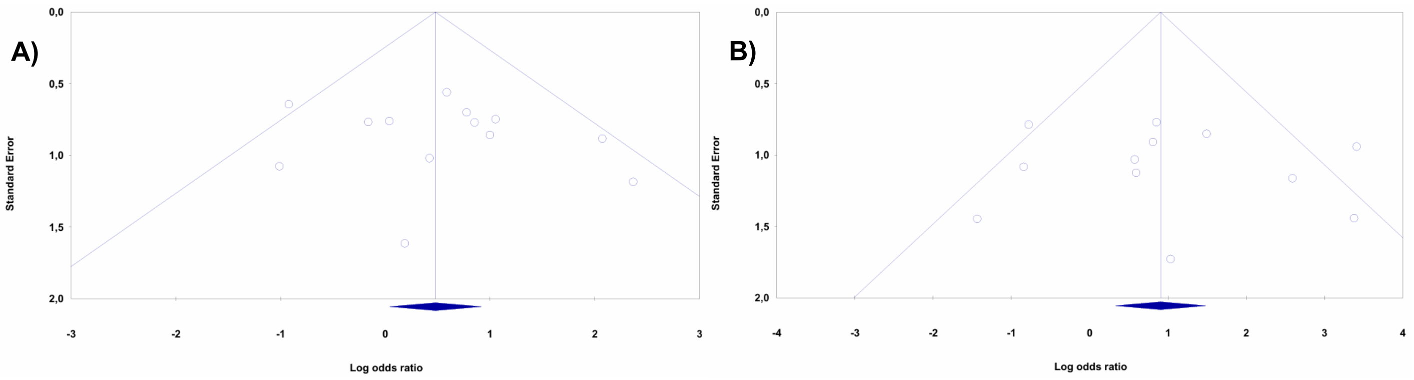

Egger’s test: 0.86; 95%CI: -2.04 to 3.76; p = 0.527

Egger’s test: 0.69; 95%CI: -3.56 to 4.95; p = 0.725

Supplementary Figure 9. Funnel plots for main meta-analyses. Association between *AR/PGR* levels in BC patients classified with IHC-surrogate subtypes Luminal A vs. Luminal B **(A)** and HER2-enriched **(B).**

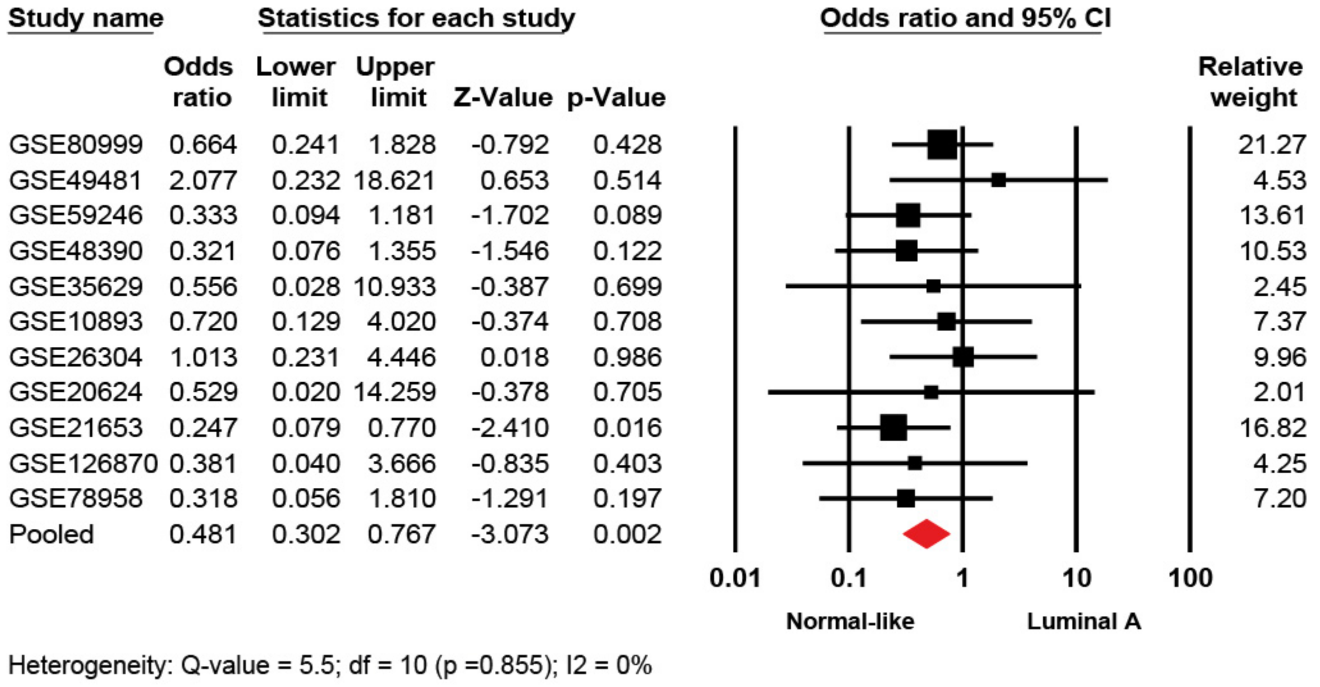

Supplementary Figure 10. High *AR/PGR* ratio is associated with Normal-like intrinsic molecular subtype - PAM50. Forest plot of Odds ratio comparing *AR/PGR* levels in BC patients classified as Normal-like vs. Luminal A. Odds ratio for each dataset are represented by the squares, and horizontal line crossing the square represents the 95% CI. The red diamonds represent the estimated overall effect.

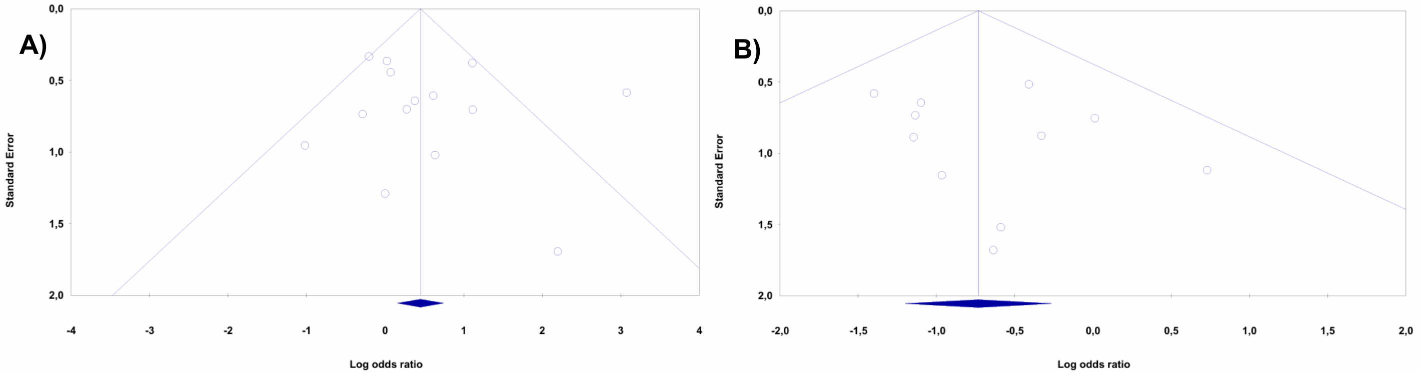

Supplementary Figure 11. Funnel plots for main meta-analyses. Association between *AR/PGR* levels in BC patients classified with intrinsic molecular subtypes HER2-enriched vs. Basal-like **(A)** and Normal-like vs. Luminal A **(B).**

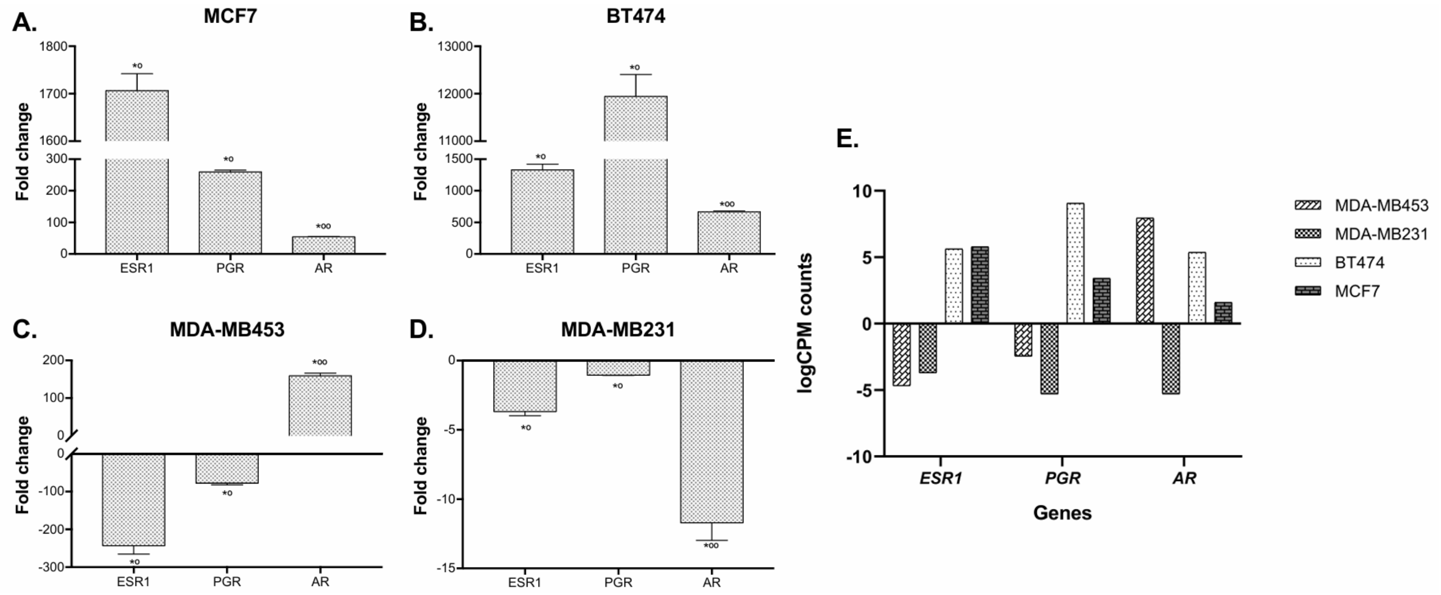

Supplementary Figure 12. qRT-PCR gene expression levels in **(A)** MCF7, **(B)** BT474, **(C)** MDA-MB453, and **(D)** MDA-MB231 cell lines. FC values were calculated relative to the GLI1 gene expression. Gene expression of each gene was compared among cell lines using the ANOVA and Tukey's post hoc test. *°° Statistical differences when compared with all cell lines. *° Statistical differences when compared with all cell lines, except for MDA-MB453 vs MDA-MB231. *Statistical differences only when MDA-MB231 was compared with all the other cell lines. **(E)** Gene expression levels in breast cancer cell lines determined by RNA-seq analysis.

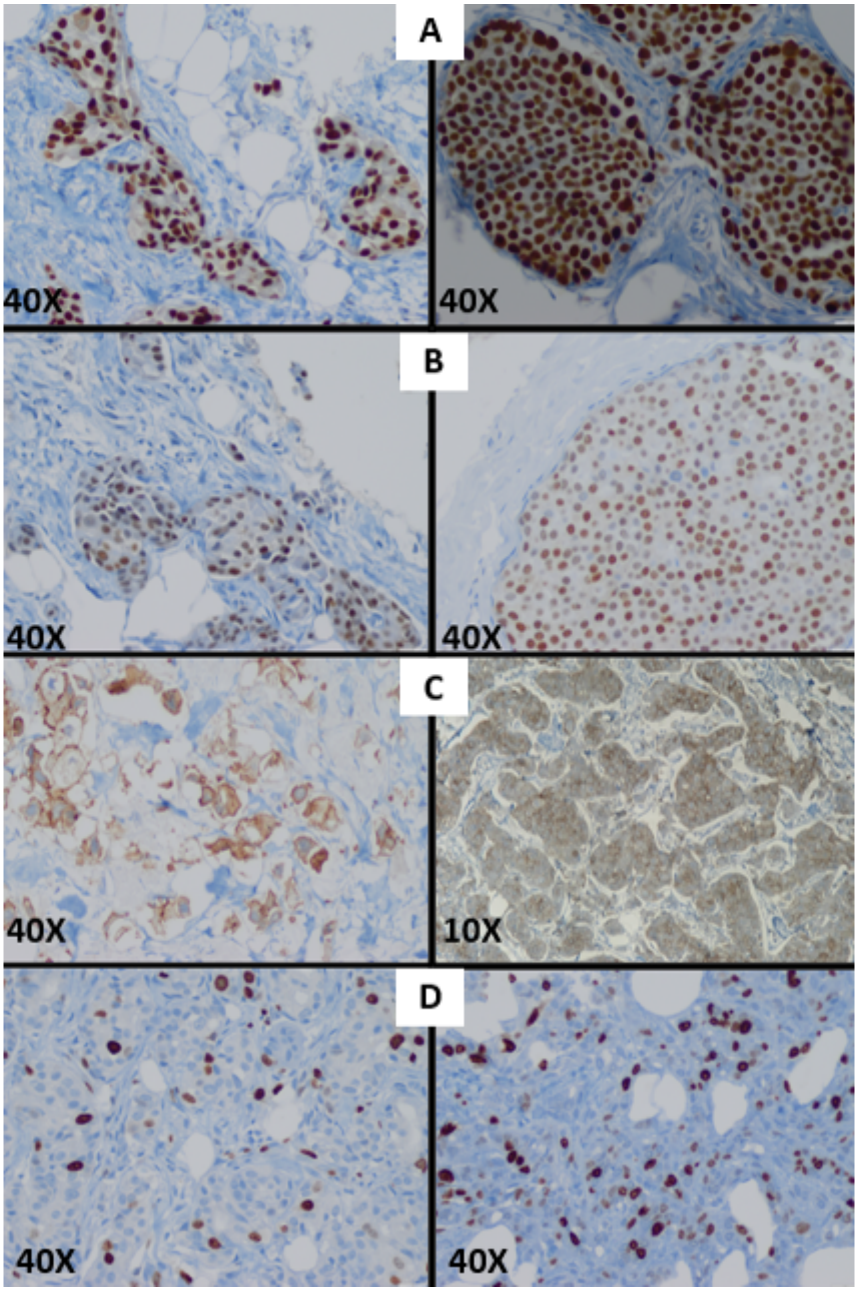

Supplementary Figure 13. Representative images of immunostaining of breast cancer tumors. ER **(A)** and PgR **(B)** positive nuclei greater than 1% were considered hormone receptor positive. According to the HER2 interpretation **(C)**, a score of 3+ was seen as positive. Determination of the Ki67 proliferation index **(D)**, left of 10% and right of 20%.

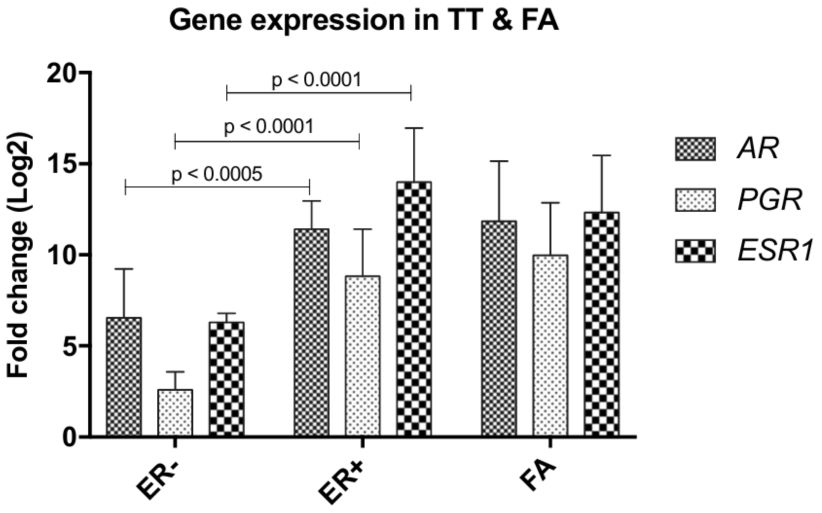

Supplementary Figure 14. mRNA gene expression determined by qRT-PCR. Data are presented as the mean expression of each gene in tumor tissue (TT) of estrogen receptor-positive (ER+) and estrogen receptor-negative and in fibroadenomas (FA) cases. The expression of each gene was significantly different ($p<0.001$) when ER- cases were compared with FA. There was not any difference ($p<0.05$) when ER+ cases were compared with FA. p values: Tukey's multiple comparisons test.
