# Supplementary material for: mRNA ratios of AR to ESR1 and PGR distinguish breast cancer subtypes based on public datasets and experimental models
Source: Sci Rep. 2025 Jul 1;15:21793. doi: 10.1038/s41598-025-06856-3 (PMC12216603; doi:10.1038/s41598-025-06856-3)
Supplement: Supplementary file 3 — Supplementary Information 3. [file 41598_2025_6856_MOESM3_ESM.docx]

**Supplementary Material**

**Supplementary Table 1.** Overview of the datasets selected from GEO for Clinical-pathological analyses.

| **GEO Dataset** | **Year** | **Country** | **Platform** | **Microarray ID** | **Reference** |
| --- | --- | --- | --- | --- | --- |
| GSE80999 | 2017 | Norway | Agilent-028004 SurePrint G3 Human GE 8x60K Microarray | GPL14550 | [1–3] |
| GSE89116 | 2016 | India | Illumina HumanHT-12 V3.0 expression beadchip | GPL6947 | [4] |
| GSE76360 | 2016 | USA | Illumina HumanHT-12 V3.0 expression beadchip | GPL6947 | [5] |
| GSE49481 | 2015 | Denmark | Agilent-02 9949 Custom SurePrint G3 Human GE 8x60K | GPL15931 | [6] |
| GSE59590 | 2015 | Italy | Illumina HumanHT-12_V3_0_R1_Bioconductor_2.11 | GPL17518 | [7] |
| GSE65194 | 2015 | France | Affymetrix Human Genome U133 Plus 2.0 Array | GPL570 | [8–10] |
| GSE60785 | 2014 | Sweden | Illumina HumanHT-12 V4.0 expression beadchip | GPL10558 | [11] |
| GSE42568 | 2013 | Ireland | Affymetrix Human Genome U133 Plus 2.0 Array | GPL570 | [12] |
| GSE37126 | 2013 | Brazil | Agilent-014850 Whole Human Genome Microarray 4x44K G4112F | GPL6480 | [13,14] |
| GSE41998 | 2013 | USA | Affymetrix Human Genome U133A 2.0 Array | GPL571 | [15] |
| GSE25307 | 2012 | Sweden | SWEGENE H_v2.1.1 55K | GPL5345 | [16] |
| GSE31364 | 2012 | Netherlands | Agendia_human_DiscoverPrint_v1 | GPL14378 | [17] |
| GSE20711 | 2011 | Canada | Affymetrix Human Genome U133 Plus 2.0 Array | GPL570 | [18] |
| GSE31863 | 2011 | Sweden | SWEGENE Human_v2.1.1 55K_condensed | GPL14374 | [19] |
| GSE24185 | 2011 | USA | Affymetrix Human Genome U133A Array | GPL96 | [20] |
| GSE22219 | 2011 | United Kingdom | Illumina humanRef-8 v1.0 expression beadchip | GPL6098 | [21] |
| GSE20194 | 2010 | China | Affymetrix Human Genome U133A Array | GPL96 | [22,23] |
| GSE10810 | 2009 | Spain | Affymetrix Human Genome U133 Plus 2.0 Array | GPL570 | [24] |
| GSE8757 | 2007 | United Kingdom | Human 30K 60-mer oligo array | GPL5737 | [25,26] |
| GSE7390 | 2007 | Canada | Affymetrix Human Genome U133A Array | GPL96 | [27,28] |
| GSE129559 | 2019 | USA | Affymetrix Human Genome U133A Array | GPL96 | [29] |
| GSE130786 | 2019 | USA | Agilent-014850 Whole Human Genome Microarray 4x44K G4112F | GPL6480 | [30] |
| GSE103744 | 2018 | Sweden | Illumina HumanHT-12 V4.0 expression beadchip | GPL10558 | [31] |
| GSE86166 | 2017 | USA | Rosetta/Merck Human RSTA Custom Affymetrix 2.0 microarray | GPL15048 | [32] |
| GSE93601 | 2017 | USA | Custom Affymetrix Human Transcriptome Array | GPL22920 | [33] |
| GSE59246 | 2016 | USA | Agilent-028004 SurePrint G3 Human GE 8x60K Microarray | GPL13607 | [34] |
| GSE71258 | 2015 | USA | Affymetrix Human Genome U133 Plus 2.0 Array | GPL570 | [35] |
| GSE61304 | 2015 | Singapore | Affymetrix Human Genome U133 Plus 2.0 Array | GPL570 | [36,37] |
| GSE29044 | 2014 | Saudi Arabia | Affymetrix Human Genome U133 Plus 2.0 Array | GPL570 | [38] |
| GSE37181 | 2014 | Italy | Illumina HumanWG-6 v3.0 expression beadchip | GPL6884 | [39] |
| GSE48390 | 2014 | Taiwan | Affymetrix Human Genome U133 Plus 2.0 Array | GPL570 | [40] |
| GSE43365 | 2013 | USA | Affymetrix Human Genome U133 Plus 2.0 Array | GPL570 | [41] |
| GSE45255 | 2013 | USA | Affymetrix Human Genome U133A Array | GPL96 | [42] |
| GSE35629 | 2013 | USA | Agilent Human 1A Oligo UNC custom Microarrays | GPL1390 | [43] |
| GSE29210 | 2013 | Canada | Agilent-014850 Whole Human Genome Microarray 4x44K G4112F | GPL6480 | [44] |
| GSE34138 | 2012 | Netherlands | Illumina HumanWG-6 v3.0 expression beadchip | GPL6884 | [45,46] |
| GSE30682 | 2012 | France | Illumina HumanWG-6 v3.0 expression beadchip | GPL6884 | [47] |
| GSE10893 | 2011 | USA | Agilent-011521 Human 1A Microarray G4110A | GPL885 | [48] |
| GSE16987 | 2011 | Canada | Illumina humanRef-8 v2.0 expression beadchip | GPL6104 | [49,50] |
| GSE22597 | 2011 | USA | Affymetrix Human Genome U133A Array | GPL96 | [51] |
| GSE26639 | 2011 | France | Affymetrix Human Genome U133 Plus 2.0 Array | GPL570 | [52] |
| GSE26304 | 2010 | Canada | Agilent-012391 Whole Human Genome Oligo Microarray G4112A | GPL6848 | [53] |
| GSE20271 | 2010 | USA | Affymetrix Human Genome U133A Array | GPL96 | [54,55] |
| GSE20624 | 2010 | USA | Agilent-011521 Human 1A Microarray G4110A | GPL1390 | [56] |
| GSE21653 | 2010 | France | Affymetrix Human Genome U133 Plus 2.0 Array | GPL570 | [57,58] |
| GSE10510 | 2008 | Germany | DKFZ Division of Molecular Genome Analysis Human Operon 4.0 oligo Array 35k | GPL6486 | [59] |
| GSE1561 | 2005 | France | Affymetrix Human Genome U133A Array | GPL96 | [60] |
| GSE2034 | 2005 | USA | Affymetrix Human Genome U133A Array | GPL96 | [61] |
| GSE17907 | 2009 | France | Affymetrix Human Genome U133 Plus 2.0 Array | GPL570 | [62] |
| GSE19615 | 2010 | USA | Affymetrix Human Genome U133 Plus 2.0 Array | GPL570 | [63] |
| GSE126870 | 2020 | United Kingdom | Illumina HumanHT-12 V4.0 expression beadchip | GPL10558 | [64] |
| GSE76727 | 2016 | United Kingdom | Illumina HumanHT-12 V4.0 expression beadchip | GPL10558 | [65] |
| GSE55639 | 2014 | Switzerland | Illumina HumanHT-12 V4.0 expression beadchip | GPL10558 | [66] |
| GSE78958 | 2016 | USA | Affymetrix Human Genome U133A 2.0 Array | GPL571 | [67] |
| GSE11121 | 2008 | Germany | Affymetrix Human Genome U133A Array | GPL96 | [68–71] |
| GSE88847 | 2019 | Canada | Affymetrix Human Gene 1.0 ST Array | GPL20171 | [72] |
| GSE4056 | 2006 | Germany | DKFZ/Operon Human Oligo Set v2.1 | GPL3367 | [73] |
| GSE102484 | 2017 | Taiwan | Affymetrix Human Genome U133 Plus 2.0 Array | GPL570 | [74] |
| GSE19697 | 2009 | USA | Affymetrix Human Genome U133 Plus 2.0 Array | GPL570 | [75] |
| GSE58812 | 2015 | France | Affymetrix Human Genome U133 Plus 2.0 Array | GPL570 | [76] |
| GSE104549 | 2018 | Italy | Illumina HumanWG-6 v3.0 expression beadchip | GPL6884 | [77] |
| GSE45827 | 2016 | France | Affymetrix Human Genome U133 Plus 2.0 Array | GPL570 | [78] |

**Supplementary Table 2.** Analysis of the association between *RA/ESR1* ratio expression values and BC classification based on molecular subtypes and IHC-surrogate subtypes.

|  |  | **Odds ratio** | **Lower Limit** | **Upper Limit** | ***p*-Value** |
| --- | --- | --- | --- | --- | --- |
| **Molecular**  **subtypes** | Basal-like vs. Luminal A | 1.336 | 0.587 | 3.042 | 0.49 |
|  | Basal-like vs. Luminal B | 1.052 | 0.385 | 2.871 | 0.922 |
|  | Basal-like vs. HER2-enriched | 0.574 | 0.342 | 0.963 | 0.035 |
|  | Basal-like vs. Normal-like | 0.486 | 0.175 | 1.346 | 0.165 |
|  | Normal-like vs. Luminal A | 0.371 | 0.145 | 0.949 | 0.039 |
|  | Normal-like vs. Luminal B | 0.437 | 0.154 | 1.241 | 0.12 |
|  | Normal-like vs. HER2-enriched | 0.942 | 0.335 | 2.646 | 0.909 |
|  | HER2-enriched vs. Luminal A | 2.366 | 0.918 | 6.099 | 0.075 |
|  | HER2-enriched vs. Luminal B | 1.896 | 0.631 | 5.7 | 0.254 |
|  | Luminal B vs. Luminal A | 1.368 | 0.867 | 2.158 | 0.179 |
| **IHC-surrogate**  **subtypes** | TNBC vs. Luminal A | 2.135 | 0.958 | 4.755 | 0.063 |
|  | TNBC vs. Luminal B | 1.542 | 0.565 | 4.207 | 0.398 |
|  | TNBC vs. HER2-enriched | 0.338 | 0.166 | 0.685 | 0.003 |
|  | HER2-enriched vs. Luminal A | 6.264 | 1.829 | 21.46 | 0.003 |
|  | HER2-enriched vs. Luminal B | 3.263 | 0.828 | 12.864 | 0.091 |
|  | Luminal B vs. Luminal A | 1.872 | 1.034 | 3.389 | 0.038 |

Abbreviations: Human epidermal growth factor receptor 2 (HER2), Immunohistochemistry (IHC), Triple Negative Breast Cancer (TNBC).

**Supplementary Table 3.** Analysis of the association between *RA/PGR* ratio expression values and BC classification based on molecular subtypes and IHC-surrogate subtypes.

|  |  | **Odds ratio** | **Lower Limit** | **Upper Limit** | ***p*-Value** |
| --- | --- | --- | --- | --- | --- |
| **Molecular**  **subtypes** | Basal-like vs. Luminal A | 1.686 | 0.637 | 4.459 | 0.293 |
|  | Basal-like vs. Luminal B | 0.833 | 0.293 | 2.369 | 0.732 |
|  | Basal-like vs. HER2-enriched | 0.891 | 0.299 | 2.655 | 0.836 |
|  | Basal-like vs. Normal-like | 0.983 | 0.354 | 2.733 | 0.974 |
|  | Normal-like vs. Luminal A | 0.481 | 0.302 | 0.767 | 0.002 |
|  | Normal-like vs. Luminal B | 0.884 | 0.48 | 1.625 | 0.69 |
|  | Normal-like vs. HER2-enriched | 0.929 | 0.379 | 2.275 | 0.871 |
|  | HER2-enriched vs. Luminal A | 1.686 | 0.637 | 4.459 | 0.293 |
|  | HER2-enriched vs. Luminal B | 0.955 | 0.373 | 2.446 | 0.924 |
|  | Luminal B vs. Luminal A | 1.695 | 1.004 | 2.836 | 0.048 |
| **IHC-surrogate**  **subtypes** | TNBC vs. Luminal A | 1.274 | 0.405 | 4.008 | 0.679 |
|  | TNBC vs. Luminal B | 0.677 | 0.193 | 2.367 | 0.541 |
|  | TNBC vs. HER2-enriched | 0.405 | 0.102 | 1.607 | 0.199 |
|  | HER2-enriched vs. Luminal A | 2.581 | 1.104 | 6.032 | 0.029 |
|  | HER2-enriched vs. Luminal B | 1.708 | 0.916 | 3.184 | 0.092 |
|  | Luminal B vs. Luminal A | 1.645 | 1.001 | 2.703 | 0.05 |

Abbreviations: Human epidermal growth factor receptor 2 (HER2), Immunohistochemistry (IHC), Triple Negative Breast Cancer (TNBC).

**References**

- 1. Aure MR, Vitelli V, Jernström S, Kumar S, Krohn M, Due EU, et al. Integrative clustering reveals a novel split in the luminal A subtype of breast cancer with impact on outcome. *Breast Cancer Res*. **2017**;19(1):44. doi: 10.1186/s13058-017-0812-y.
  2. Knutsen E, Lellahi SM, Aure MR, Nord S, Fismen S, Larsen KB, et al. The expression of the long NEAT1_2 isoform is associated with human epidermal growth factor receptor 2-positive breast cancers. *Sci Rep*. **2020** Jan 28;10(1):1277. doi: 10.1038/s41598-020-57759-4.
  3. Pladsen A V, Nilsen G, Rueda OM, Aure MR, Borgan Ø, Liestøl K, et al. DNA copy number motifs are strong and independent predictors of survival in breast cancer. *Commun Biol*. **2020**;3(1):153. doi: 10.1038/s42003-020-0884-6.
  4. Malvia S, Bagadi SAR, Pradhan D, Chintamani C, Bhatnagar A, Arora D, et al. Study of Gene Expression Profiles of Breast Cancers in Indian Women. *Sci Rep*.
  5. Varadan V, Gilmore H, Miskimen KLS, Tuck D, Parsai S, Awadallah A, et al. Immune Signatures Following Single Dose Trastuzumab Predict Pathologic Response to PreoperativeTrastuzumab and Chemotherapy in HER2-Positive Early Breast Cancer. *Clin Cancer Res*. **2016**;22(13):3249–59. doi: 10.1158/1078- 0432.CCR-15-2021.
  6. Larsen MJ, Thomassen M, Tan Q, Lænkholm A-V, Bak M, Sørensen KP, et al. RNA profiling reveals familial aggregation of molecular subtypes in non-BRCA1/2 breast cancer families. *BMC Med Genomics*. **2014** Jan 31;7:9. doi: 10.1186/1755-8794-7-9.
  7. Huang X, Dugo M, Callari M, Sandri M, De Cecco L, Valeri B, et al. Molecular portrait of breast cancer in China reveals comprehensive transcriptomic likeness to Caucasian breast cancer and low prevalence of luminal A subtype. *Cancer Med*. **2015** Jul;4(7):1016–30. doi: 10.1002/cam4.442.
  8. Maubant S, Tesson B, Maire V, Ye M, Rigaill G, Gentien D, et al. Transcriptome analysis of Wnt3a-treated triple-negative breast cancer cells. *PLoS One*.
  9. Maire V, Baldeyron C, Richardson M, Tesson B, Vincent-Salomon A, Gravier E, et al. TTK/hMPS1 is an attractive therapeutic target for triple-negative breast cancer. *PLoS One*. **2013**;8(5):e63712. doi: 10.1371/journal.pone.0063712.
  10. Maire V, Némati F, Richardson M, Vincent-Salomon A, Tesson B, Rigaill G, et al. Polo-like kinase 1: a potential therapeutic option in combination with conventional chemotherapy for the management of patients with triple-negative breast cancer. *Cancer Res*. **2013** Jan 15;73(2):813–23. doi: 10.1158/0008-5472.CAN- 12-2633.
  11. Saal LH, Vallon-Christersson J, Häkkinen J, Hegardt C, Grabau D, Winter C, et al. The Sweden Cancerome Analysis Network - Breast (SCAN-B) Initiative: a large- scale multicenter infrastructure towards implementation of breast cancer genomic analyses in the clinical routine. *Genome Med*. **2015**;7(1):20. doi: 10.1186/s13073- 015-0131-9.
  12. Clarke C, Madden SF, Doolan P, Aherne ST, Joyce H, O’Driscoll L, et al. Correlating transcriptional networks to breast cancer survival: a large-scale coexpression analysis. *Carcinogenesis*. **2013** Oct;34(10):2300–8. doi: 10.1093/carcin/bgt208.
  13. Carraro DM, Koike Folgueira MAA, Garcia Lisboa BC, Ribeiro Olivieri EH, Vitorino Krepischi AC, de Carvalho AF, et al. Comprehensive analysis of BRCA1, BRCA2 and TP53 germline mutation and tumor characterization: a portrait of early-onset breast cancer in Brazil. *PLoS One*. **2013**;8(3):e57581. doi: 10.1371/journal.pone.0057581.
  14. Bastos EP, Brentani H, Pasini FS, Silva ART, Torres CH, Puga RD, et al. MicroRNAs discriminate familial from sporadic non-BRCA1/2 breast carcinoma arising in patients ≤35 years. *PLoS One*. **2014**;9(7):e101656. doi: 10.1371/journal.pone.0101656.
  15. Horak CE, Pusztai L, Xing G, Trifan OC, Saura C, Tseng L-M, et al. Biomarker analysis of neoadjuvant doxorubicin/cyclophosphamide followed by ixabepilone or Paclitaxel in early-stage breast cancer. *Clin Cancer Res*. **2013** Mar 15;19(6):1587–95. doi: 10.1158/1078-0432.CCR-12-1359.
  16. Jönsson G, Staaf J, Vallon-Christersson J, Ringnér M, Gruvberger-Saal SK, Saal LH, et al. The retinoblastoma gene undergoes rearrangements in BRCA1-deficient basal-like breast cancer. *Cancer Res*. **2012** Aug 15;72(16):4028–36. doi: 10.1158/0008-5472.CAN-12-0097.
  17. Molloy TJ, Roepman P, Naume B, van’t Veer LJ. A prognostic gene expression profile that predicts circulating tumor cell presence in breast cancer patients. *PLoS One*. **2012**;7(2):e32426. doi: 10.1371/journal.pone.0032426.
  18. Dedeurwaerder S, Desmedt C, Calonne E, Singhal SK, Haibe-Kains B, Defrance M, et al. DNA methylation profiling reveals a predominant immune component in breast cancers. *EMBO Mol Med*. **2011** Dec;3(12):726–41. doi: 10.1002/emmm.201100801.
  19. Niméus-Malmström E, Krogh M, Malmström P, Strand C, Fredriksson I, Karlsson P, et al. Gene expression profiling in primary breast cancer distinguishes patients developing local recurrence after breast-conservation surgery, with or without postoperative radiotherapy. *Breast Cancer Res*. **2008**;10(2):R34. doi: 10.1186/bcr1997.
  20. Creighton CJ, Sada YH, Zhang Y, Tsimelzon A, Wong H, Dave B, et al. A gene transcription signature of obesity in breast cancer. *Breast Cancer Res Treat*. **2012**.
  21. Buffa FM, Camps C, Winchester L, Snell CE, Gee HE, Sheldon H, et al. microRNA-associated progression pathways and potential therapeutic targets identified by integrated mRNA and microRNA expression profiling in breast cancer. *Cancer Res*. **2011** Sep 1;71(17):5635–45. doi: 10.1158/0008-5472.CAN-11-0489.
  22. Popovici V, Chen W, Gallas BG, Hatzis C, Shi W, Samuelson FW, et al. Effect of training-sample size and classification difficulty on the accuracy of genomic predictors. *Breast Cancer Res*. **2010**;12(1):R5. doi: 10.1186/bcr2468.
  23. Shi L, Campbell G, Jones WD, Campagne F, Wen Z, Walker SJ, et al. The MicroArray Quality Control (MAQC)-II study of common practices for the development and validation of microarray-based predictive models. *Nat Biotechnol*. **2010** Aug;28(8):827–38. doi: 10.1038/nbt.1665.
  24. Pedraza V, Gomez-Capilla JA, Escaramis G, Gomez C, Torné P, Rivera JM, et al. Gene expression signatures in breast cancer distinguish phenotype characteristics, histologic subtypes, and tumor invasiveness. *Cancer*. **2010** Jan 15;116(2):486–96. doi: 10.1002/cncr.24805.
  25. Chin SF, Teschendorff AE, Marioni JC, Wang Y, Barbosa-Morais NL, Thorne NP, et al. High-resolution aCGH and expression profiling identifies a novel genomic subtype of ER negative breast cancer. *Genome Biol*. **2007**;8(10):R215. doi: 10.1186/gb-2007-8-10-r215.
  26. Zhang Y, Martens JWM, Yu JX, Jiang J, Sieuwerts AM, Smid M, et al. Copy number alterations that predict metastatic capability of human breast cancer. *Cancer Res*. **2009** May 1;69(9):3795–801. doi: 10.1158/0008-5472.CAN-08-4596.
  27. Desmedt C, Piette F, Loi S, Wang Y, Lallemand F, Haibe-Kains B, et al. Strong time dependence of the 76-gene prognostic signature for node-negative breast cancer patients in the TRANSBIG multicenter independent validation series. *Clin Cancer Res*. **2007** Jun 1;13(11):3207–14. doi: 10.1158/1078-0432.CCR-06-2765.
  28. Patil P, Bachant-Winner P-O, Haibe-Kains B, Leek JT. Test set bias affects reproducibility of gene signatures. *Bioinformatics*. **2015** Jul 15;31(14):2318–23. doi: 10.1093/bioinformatics/btv157.
  29. Sinn B V, Fu C, Lau R, Litton J, Tsai T-H, Murthy R, et al. SETER/PR: a robust 18-gene predictor for sensitivity to endocrine therapy for metastatic breast cancer.
  30. Zoeller JJ, F. Press M, Selfors LM, Dering J, Slamon DJ, Hurvitz SA, et al. Clinical evaluation of BCL-2/XL levels pre- and post- HER2-targeted therapy. *PLoS One*.
  31. Sjöström M, Staaf J, Edén P, Wärnberg F, Bergh J, Malmström P, et al. Identification and validation of single-sample breast cancer radiosensitivity gene expression predictors. *Breast Cancer Res*. **2018**;20(1):64. doi: 10.1186/s13058-018-0978-y.
  32. Prabhakaran S, Rizk VT, Ma Z, Cheng C-H, Berglund AE, Coppola D, et al. Evaluation of invasive breast cancer samples using a 12-chemokine gene expression score: correlation with clinical outcomes. *Breast Cancer Res*. **2017**;19(1):71. doi: 10.1186/s13058-017-0864-z.
  33. Wang J, Zhang X, Beck AH, Collins LC, Chen WY, Tamimi RM, et al. Alcohol Consumption and Risk of Breast Cancer by Tumor Receptor Expression. *Horm Cancer*. **2015** Dec;6(5–6):237–46. doi: 10.1007/s12672-015-0235-0.
  34. Lesurf R, Aure MR, Mørk HH, Vitelli V, Oslo Breast Cancer Research Consortium (OSBREAC), Lundgren S, et al. Molecular Features of Subtype-Specific Progression from Ductal Carcinoma In Situ to Invasive Breast Cancer. *Cell Rep*. **2016**;16(4):1166–79. doi: 10.1016/j.celrep.2016.06.051.
  35. Xiang J, Hurchla MA, Fontana F, Su X, Amend SR, Esser AK, et al. CXCR4 Protein Epitope Mimetic Antagonist POL5551 Disrupts Metastasis and Enhances Chemotherapy Effect in Triple-Negative Breast Cancer. *Mol Cancer Ther*. **2015** Nov;14(11):2473–85. doi: 10.1158/1535-7163.MCT-15-0252.
  36. Grinchuk O V, Motakis E, Yenamandra SP, Ow GS, Jenjaroenpun P, Tang Z, et al. Sense-antisense gene-pairs in breast cancer and associated pathological pathways. *Oncotarget*. **2015** Dec 8;6(39):42197–221. doi: 10.18632/oncotarget.6255.
  37. Aswad L, Yenamandra SP, Ow GS, Grinchuk O, Ivshina A V, Kuznetsov VA. Genome and transcriptome delineation of two major oncogenic pathways governing invasive ductal breast cancer development. *Oncotarget*. **2015** Nov 3;6(34):36652–74. doi: 10.18632/oncotarget.5543.
  38. Colak D, Nofal A, Albakheet A, Nirmal M, Jeprel H, Eldali A, et al. Age-specific gene expression signatures for breast tumors and cross-species conserved potential cancer progression markers in young women. *PLoS One*. **2013**;8(5):e63204. doi: 10.1371/journal.pone.0063204.
  39. Callari M, Musella V, Di Buduo E, Sensi M, Miodini P, Dugo M, et al. Subtype-dependent prognostic relevance of an interferon-induced pathway metagene in node-negative breast cancer. *Mol Oncol*. **2014** Oct;8(7):1278–89. doi: 10.1016/j.molonc.2014.04.010.
  40. Huang C-C, Tu S-H, Lien H-H, Jeng J-Y, Huang C-S, Huang C-J, et al. Concurrent gene signatures for han chinese breast cancers. *PLoS One*. **2013**;8(10):e76421. doi: 10.1371/journal.pone.0076421.
  41. Metzger-Filho O, Catteau A, Michiels S, Buyse M, Ignatiadis M, Saini KS, et al. Genomic Grade Index (GGI): feasibility in routine practice and impact on treatment decisions in early breast cancer. *PLoS One*. **2013**;8(8):e66848. doi: 10.1371/journal.pone.0066848.
  42. Nagalla S, Chou JW, Willingham MC, Ruiz J, Vaughn JP, Dubey P, et al. Interactions between immunity, proliferation and molecular subtype in breast cancer prognosis. *Genome Biol*. **2013** Apr 29;14(4):R34. doi: 10.1186/gb-2013-14-4-r34.
  43. Ulirsch J, Fan C, Knafl G, Wu MJ, Coleman B, Perou CM, et al. Vimentin DNA methylation predicts survival in breast cancer. *Breast Cancer Res Treat*. **2013**
  44. Bastani M, Vos L, Asgarian N, Deschenes J, Graham K, Mackey J, et al. A machine learned classifier that uses gene expression data to accurately predict estrogen receptor status. *PLoS One*. **2013**;8(12):e82144. doi: 10.1371/journal.pone.0082144.
  45. de Ronde JJ, Lips EH, Mulder L, Vincent AD, Wesseling J, Nieuwland M, et al. SERPINA6, BEX1, AGTR1, SLC26A3, and LAPTM4B are markers of resistance to neoadjuvant chemotherapy in HER2-negative breast cancer. *Breast Cancer Res Treat*. **2013** Jan;137(1):213–23. doi: 10.1007/s10549-012-2340-x.
  46. Kersten K, Coffelt SB, Hoogstraat M, Verstegen NJM, Vrijland K, Ciampricotti M, et al. Mammary tumor-derived CCL2 enhances pro-metastatic systemic inflammation through upregulation of IL1β in tumor-associated macrophages. *Oncoimmunology*. 6(8):e1334744. doi: 10.1080/2162402X.2017.1334744.
  47. Servant N, Bollet MA, Halfwerk H, Bleakley K, Kreike B, Jacob L, et al. Search for a gene expression signature of breast cancer local recurrence in young women.
  48. Weigman VJ, Chao H-H, Shabalin AA, He X, Parker JS, Nordgard SH, et al. Basal-like Breast cancer DNA copy number losses identify genes involved in genomic instability, response to therapy, and patient survival. *Breast Cancer Res Treat*. **2012** Jun;133(3):865–80. doi: 10.1007/s10549-011-1846-y.
  49. Wang D-Y, Done SJ, McCready DR, Boerner S, Kulkarni S, Leong WL. A new gene expression signature, the ClinicoMolecular Triad Classification, may improve prediction and prognostication of breast cancer at the time of diagnosis. *Breast Cancer Res*. **2011** Sep 22;13(5):R92. doi: 10.1186/bcr3017.
  50. Wang D-Y, Done SJ, Mc Cready DR, Leong WL. Validation of the prognostic gene portfolio, ClinicoMolecular Triad Classification, using an independent prospective breast cancer cohort and external patient populations. *Breast Cancer Res*. **2014** Jul 4;16(4):R71. doi: 10.1186/bcr3686.
  51. Iwamoto T, Bianchini G, Qi Y, Cristofanilli M, Lucci A, Woodward WA, et al. Different gene expressions are associated with the different molecular subtypes of inflammatory breast cancer. *Breast Cancer Res Treat*. **2011** Feb;125(3):785–95. doi: 10.1007/s10549-010-1280-6.
  52. de Cremoux P, Valet F, Gentien D, Lehmann-Che J, Scott V, Tran-Perennou C, et al. Importance of pre-analytical steps for transcriptome and RT-qPCR analyses in the context of the phase II randomised multicentre trial REMAGUS02 of neoadjuvant chemotherapy in breast cancer patients. *BMC Cancer*. **2011** Jun 1;11:215. doi: 10.1186/1471-2407-11-215.
  53. Muggerud AA, Hallett M, Johnsen H, Kleivi K, Zhou W, Tahmasebpoor S, et al. Molecular diversity in ductal carcinoma in situ (DCIS) and early invasive breast cancer. *Mol Oncol*. **2010** Aug;4(4):357–68. doi: 10.1016/j.molonc.2010.06.007.
  54. Tabchy A, Valero V, Vidaurre T, Lluch A, Gomez H, Martin M, et al. Evaluation of a 30-gene paclitaxel, fluorouracil, doxorubicin, and cyclophosphamide chemotherapy response predictor in a multicenter randomized trial in breast cancer. *Clin Cancer Res*. **2010** Nov 1;16(21):5351–61. doi: 10.1158/1078-0432.CCR-10- 1265.
  55. Shen K, Song N, Kim Y, Tian C, Rice SD, Gabrin MJ, et al. A systematic evaluation of multi-gene predictors for the pathological response of breast cancer patients to chemotherapy. *PLoS One*. **2012**;7(11):e49529. doi: 10.1371/journal.pone.0049529.
  56. Anders CK, Fan C, Parker JS, Carey LA, Blackwell KL, Klauber-DeMore N, et al. Breast carcinomas arising at a young age: unique biology or a surrogate for aggressive intrinsic subtypes? *J Clin Oncol*. **2011** Jan 1;29(1):e18-20. doi: 10.1200/JCO.2010.28.9199.
  57. Sabatier R, Finetti P, Cervera N, Lambaudie E, Esterni B, Mamessier E, et al. A gene expression signature identifies two prognostic subgroups of basal breast cancer. *Breast Cancer Res Treat*. **2011** Apr;126(2):407–20. doi: 10.1007/s10549-010-0897-9.
  58. Sabatier R, Finetti P, Adelaide J, Guille A, Borg J-P, Chaffanet M, et al. Down-regulation of ECRG4, a candidate tumor suppressor gene, in human breast cancer.
  59. Calabrò A, Beissbarth T, Kuner R, Stojanov M, Benner A, Asslaber M, et al. Effects of infiltrating lymphocytes and estrogen receptor on gene expression and prognosis in breast cancer. *Breast Cancer Res Treat*. **2009** Jul;116(1):69–77. doi: 10.1007/s10549-008-0105-3.
  60. Farmer P, Bonnefoi H, Becette V, Tubiana-Hulin M, Fumoleau P, Larsimont D, et al. Identification of molecular apocrine breast tumours by microarray analysis.
  61. Wang Y, Klijn JGM, Zhang Y, Sieuwerts AM, Look MP, Yang F, et al. Gene-expression profiles to predict distant metastasis of lymph-node-negative primary breast cancer. *Lancet (London, England)*. 365(9460):671–9. doi: 10.1016/S0140-6736(05)17947-1.
  62. Sircoulomb F, Bekhouche I, Finetti P, Adélaïde J, Ben Hamida A, Bonansea J, et al. Genome profiling of ERBB2-amplified breast cancers. *BMC Cancer*. **2010** Oct 8;10:539. doi: 10.1186/1471-2407-10-539.
  63. Li Y, Zou L, Li Q, Haibe-Kains B, Tian R, Li Y, et al. Amplification of LAPTM4B and YWHAZ contributes to chemotherapy resistance and recurrence of breast cancer. *Nat Med*. **2010** Feb;16(2):214–8. doi: 10.1038/nm.2090.
  64. Gao Q, López-Knowles E, Cheang MCU, Morden J, Ribas R, Sidhu K, et al. Impact of aromatase inhibitor treatment on global gene expression and its association with antiproliferative response in ER+ breast cancer in postmenopausal patients. *Breast Cancer Res*. **2019**;22(1):2. doi: 10.1186/s13058-019-1223-z.
  65. Pearce DA, Arthur LM, Turnbull AK, Renshaw L, Sabine VS, Thomas JS, et al. Tumour sampling method can significantly influence gene expression profiles derived from neoadjuvant window studies. *Sci Rep*. **2016**;6:29434. doi: 10.1038/srep29434.
  66. Piscuoglio S, Ng CKY, Martelotto LG, Eberle CA, Cowell CF, Natrajan R, et al. Integrative genomic and transcriptomic characterization of papillary carcinomas of the breast. *Mol Oncol*. **2014** Dec;8(8):1588–602. doi: 10.1016/j.molonc.2014.06.011.
  67. Toro AL, Costantino NS, Shriver CD, Ellsworth DL, Ellsworth RE. Effect of obesity on molecular characteristics of invasive breast tumors: gene expression analysis in a large cohort of female patients. *BMC Obes*. **2016**;3:22. doi: 10.1186/s40608-016-0103-7.
  68. Schmidt M, Böhm D, von Törne C, Steiner E, Puhl A, Pilch H, et al. The humoral immune system has a key prognostic impact in node-negative breast cancer.
  69. Cadenas C, van de Sandt L, Edlund K, Lohr M, Hellwig B, Marchan R, et al. Loss of circadian clock gene expression is associated with tumor progression in breast cancer. *Cell Cycle*. **2014**;13(20):3282–91. doi: 10.4161/15384101.2014.954454.
  70. Hellwig B, Hengstler JG, Schmidt M, Gehrmann MC, Schormann W, Rahnenführer J. Comparison of scores for bimodality of gene expression distributions and genome-wide evaluation of the prognostic relevance of high-scoring genes. *BMC Bioinformatics*. **2010** May 25;11:276. doi: 10.1186/1471-2105-11-276.
  71. Heimes A-S, Härtner F, Almstedt K, Krajnak S, Lebrecht A, Battista MJ, et al. Prognostic Significance of Interferon-γ and Its Signaling Pathway in Early Breast Cancer Depends on the Molecular Subtypes. *Int J Mol Sci*. **2020** Sep 29;21(19). doi: 10.3390/ijms21197178.
  72. Gruosso T, Gigoux M, Manem VSK, Bertos N, Zuo D, Perlitch I, et al. Spatially distinct tumor immune microenvironments stratify triple-negative breast cancers. *J Clin Invest*. **2019**;129(4):1785–800. doi: 10.1172/JCI96313.
  73. Thuerigen O, Schneeweiss A, Toedt G, Warnat P, Hahn M, Kramer H, et al. Gene expression signature predicting pathologic complete response with gemcitabine, epirubicin, and docetaxel in primary breast cancer. *J Clin Oncol*. **2006** Apr 20;24(12):1839–45. doi: 10.1200/JCO.2005.04.7019.
  74. Cheng SH-C, Huang T-T, Cheng Y-H, Tan TBK, Horng C-F, Wang YA, et al. Validation of the 18-gene classifier as a prognostic biomarker of distant metastasis in breast cancer. *PLoS One*. **2017**;12(9):e0184372. doi: 10.1371/journal.pone.0184372.
  75. Lin Y, Lin S, Watson M, Trinkaus KM, Kuo S, Naughton MJ, et al. A gene expression signature that predicts the therapeutic response of the basal-like breast cancer to neoadjuvant chemotherapy. *Breast Cancer Res Treat*. **2010** Oct;123(3):691–9. doi: 10.1007/s10549-009-0664-y.
  76. Jézéquel P, Loussouarn D, Guérin-Charbonnel C, Campion L, Vanier A, Gouraud W, et al. Gene-expression molecular subtyping of triple-negative breast cancer tumours: importance of immune response. *Breast Cancer Res*. **2015** Mar 20;17:43. doi: 10.1186/s13058-015-0550-y.
  77. Cappelletti V, Iorio E, Miodini P, Silvestri M, Dugo M, Daidone MG. Metabolic Footprints and Molecular Subtypes in Breast Cancer. *Dis Markers*.
  78. Gruosso T, Mieulet V, Cardon M, Bourachot B, Kieffer Y, Devun F, et al. Chronic oxidative stress promotes H2AX protein degradation and enhances chemosensitivity in breast cancer patients. *EMBO Mol Med*. **2016**;8(5):527–49. doi: 10.15252/emmm.201505891.
